# Exposure to antibiotics during pregnancy or early infancy and the risk of autoimmune disease in children: A nationwide cohort study in Korea

**DOI:** 10.1371/journal.pmed.1004677

**Published:** 2025-08-21

**Authors:** Eun-Young Choi, Sungho Bea, Hyesung Lee, Ahhyung Choi, Jung Yeol Han, Eun Ha Kang, Ju-Young Shin

**Affiliations:** 1 School of Pharmacy, Sungkyunkwan University, Suwon, South Korea; 2 Division of Pharmacoepidemiology and Pharmacoeconomics, Department of Medicine, Brigham and Women’s Hospital and Harvard Medical School, Boston, Massachusetts, United States of America; 3 Department of Medical Informatics, Kangwon National University College of Medicine, Chuncheon, South Korea; 4 Korean Mothersafe Counselling Center, Department of Obstetrics and Gynecology, Inje University Ilsan Paik Hospital, Goyang, South Korea; 5 Division of Rheumatology, Seoul National University College of Medicine, Seoul National University Bundang Hospital, Seongnam, South Korea; 6 Department Biohealth Regulatory Science, Sungkyunkwan University, Suwon, South Korea; 7 Department of Clinical Research Design & Evaluation, Samsung Advanced Institute for Health Sciences and Technology (SAIHST), Sungkyunkwan University, Seoul, South Korea; London School of Hygiene and Tropical Medicine, UNITED KINGDOM OF GREAT BRITAIN AND NORTHERN IRELAND

## Abstract

**Background:**

Emerging evidence suggests that prenatal or early-life exposure to antibiotics may contribute to the development of autoimmune diseases in children. However, previous studies investigating this association have yielded conflicting and inconclusive results, partly due to challenges related to confounding by indication and underlying genetic or familial factors.

**Methods and findings:**

A nationwide cohort study was conducted using mother-child linked claims database of Korea’s National Health Insurance Service between 2008 and 2021. Among individuals with diagnosis of infections, children exposed to antibiotics during pregnancy or early infancy were compared to those who were not exposed. The autoimmune-related outcomes of interest were the onset of type 1 diabetes, juvenile idiopathic arthritis, inflammatory bowel disease (ulcerative colitis, Crohn’s disease), systemic lupus erythematosus, and Hashimoto’s thyroiditis. The antibiotic-exposed pregnancies were compared to unexposed pregnancies using inverse probability of treatment weighting (IPTW) methods to adjust for potential imbalances and confounding by indication. Also, sibling-matched analyses were performed to minimize bias from within-family confounders. Cox proportional hazard model was applied to assess associations, and clinically relevant subgroup analyses, including sex, subclasses and exposed timing of antibiotics were also conducted. Before IPTW, we identified 1,516,574 exposed children and 1,186,516 unexposed children for pregnancy analysis, and 1,925,585 exposed and 1,421,464 unexposed for the infancy analysis. In the pregnancy analysis within the infection-restricted population, IPTW analyses showed null association between antibiotic exposure and autoimmune diseases, including type 1 diabetes (IPTW HR 1.14, 95% CI [0.96, 1.35], *p*-value = 0.132), juvenile idiopathic arthritis (HR 1.02, 95% CI [0.85, 1.22], *p*-value = 0.830), ulcerative colitis (HR 1.02, 95% CI [0.76, 1.37], *p*-value = 0.895), Crohn’s disease (HR 1.16, 95% CI [0.98, 1.36], *p*-value = 0.076), systemic lupus erythematosus (HR 0.70, 95% CI [0.49, 1.01], *p*-value = 0.053), and Hashimoto’s thyroiditis (HR 1.06, 95% CI [0.91, 1.23], *p*-value = 0.448). In the infancy analysis within the infection-restricted population, IPTW analyses showed no substantial differences in autoimmune disease risk between exposed and non-exposed groups for type 1 diabetes (IPTW HR 1.05, 95% CI [0.88, 1.26], *p*-value = 0.594) and juvenile idiopathic arthritis (HR 1.11, 95% CI [0.93, 1.33], *p*-value = 0.253), ulcerative colitis (HR 0.95, 95% CI [0.67, 1.36], *p*-value = 0.776), Crohn’s disease (HR 1.07, 95% CI [0.91, 1.25], *p*-value = 0.403), systemic lupus erythematosus (HR 1.46, 95% CI [0.95, 2.26], *p*-value = 0.087), and Hashimoto’s thyroiditis (HR 1.14, 95% CI [0.97, 1.33], *p*-value = 0.104). These results also showed similar associations in sibling-matched analyses. Subgroup analyses showed that maternal use of cephalosporins or antibiotics during first or second trimester was associated with a small increased risk of Crohn’s disease in pregnancy analysis, while antibiotic exposure in males or during the first two months of life was associated with a modestly increased risk of autoimmune thyroiditis in infancy analysis. The primary limitations of this study include potential residual confounding due to unmeasured variables.

**Conclusions:**

In this nationwide cohort study, we found no association between early-life antibiotic exposure and the overall risk of autoimmune diseases in children. These findings underscore the importance of ensuring that antibiotic use during pregnancy and early infancy is guided by clear clinical indications and highlight the need for further research to explore subgroup-specific risks in greater detail.

## Author summary

**Why was this study done?:**

- Emerging evidence suggests that prenatal or early life exposure to antibiotics could be a contributing factor for the development of autoimmune diseases among children.
- Previous studies on the association between antibiotic use and autoimmune disease report conflicting results, and these studies were limited by potential confounding due to genetic factors or indications.

**What did the researchers do and find?:**

- In this nationwide mother–child linked cohort study, we investigated the association between antibiotic exposure during pregnancy and early infancy and the risk of autoimmune diseases, restricting the analysis to patients with infections and using inverse probability of treatment weighting and sibling-matched analyses to mitigate confounding.
- Overall, there was no association between antibiotic exposure during pregnancy or early infancy and the overall incidence of autoimmune diseases in children.

**What do these findings mean?:**

- Early-life antibiotic exposure was not associated with an increased risk of autoimmune diseases in children, indicating that the potential therapeutic benefits of antibiotic use may outweigh the minimal risk of autoimmune outcomes in these periods.
- The principal limitations of the study relate to confounding by the presence of unmeasured factors that could not be addressed through available data.

## Abstract 3

In a nationwide Korean cohort study, Ju-Young Shin and colleagues investigated whether there is an association between early life exposure to antibiotics and the development of autoimmune diseases.

## Introduction

The incidence of autoimmune diseases has seen a marked increase over the past few decades, particularly in pediatric populations [1,2]. This trend has been observed globally, with variations in incidence influenced by genetic predisposition, environmental factors, and healthcare practices across different regions [1,3]. Given their long life expectancy, children with autoimmune diseases often face a substantial burdened of chronic illness, which can severely impact their quality of life [4–6]. While the precise etiology of these widespread autoimmune conditions remains unclear, emerging evidence suggests that prenatal or early life exposure to antibiotics could be a contributing factor [7,8]. This hypothesis is based on the understanding that antibiotics, while essential for managing bacterial infections, can disrupt the development of immune system and microbiome, potentially predisposing individuals to autoimmune disorders [8–10]. As antibiotic exposure during the formative years of the immune system can significantly impact children’s immune development, it is essential to consider the long-term consequences of antibiotic use during this critical period for the establishment of lifelong immunity and gut microbiome [11].

Antibiotics are commonly prescribed during pregnancy for infections such as respiratory or urinary tract infections, accounting for about 80% of prescriptions [12,13]. Similarly, during infancy, antibiotics are the most frequently prescribed medications [14]. While previous studies have examined how antibiotic exposure relates to autoimmune disease in children, they often do not fully consider the influence of infection or familial predispositions, which typically prompts antibiotic use and can greatly affect autoimmune disease development [15–24]. Additionally, the evidence on whether antibiotic use increases the risk of autoimmune diseases among children is conflicting and warrants further research.

Therefore, we aimed to investigate the association between antibiotic exposure during pregnancy and early infancy and the risk of autoimmune diseases. To mitigate potential confounding factors, we also restricted the target population to those diagnosed with infections and applied stabilized inverse probability of treatment weighting (IPTW) and sibling-matching approaches.

## Methods

### Data source

We conducted a retrospective cohort study utilizing data from the South Korea National Health Insurance Service–National Health Insurance Database (NHIS–NHID). By utilizing unique health insurance card numbers shared within families and cross-referencing them with delivery dates, the NHIS accurately identified maternal-child pairs through a validated linkage algorithm [25,26]. The NHIS–NHID database provides near-complete coverage of the Korean population, as South Korea operates a single-payer, universal healthcare system in which nearly all residents (97%) are enrolled. This ensures that the data captures virtually all healthcare utilization in Korea, including inpatient, outpatient, and public health center services, minimizing selection bias related to healthcare access. Socioeconomic background is accounted for using income-level indicators derived from health insurance contributions, which serve as reliable proxies for income level. Additionally, given that Korea’s single-payer system ensures relatively uniform access to healthcare services, and supplementary policies targeting pregnant women and children further minimize disparities in provider access across income levels. The NHIS–NHID includes comprehensive data on sociodemographic factors, inpatient and outpatient healthcare services (including diagnoses, procedures, and medication prescriptions), health examination records for both mothers and their children, and vital statistics, which are linked with data from Statistics Korea. The requirement for informed consent was waived because personal codes are deidentified in the database. The protocol for this study was reviewed and approved by the institutional review board of Sungkyunkwan University (2024-04-060). All analyses were conducted in accordance with this protocol (S1 Protocol) and reported following the Strengthening the Reporting of Observational Studies in Epidemiology (STROBE) guidelines (S1 STROBE Checklist).

### Cohorts for pregnancy and infancy analyses

**Fig 1:**
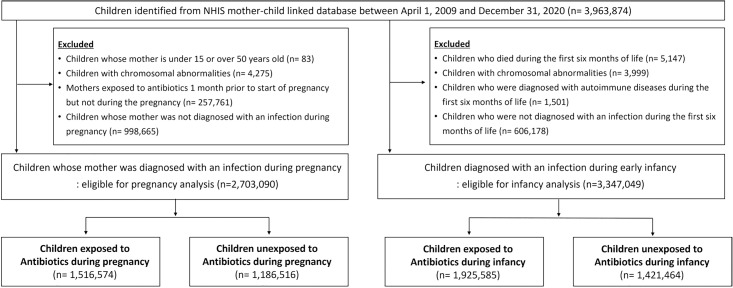
Study flow chart for two distinctive cohort construction. Abbreviations: NHIS, National Health Insurance Service.

Two distinct cohorts were constructed for all children born between April 1, 2009, and December 31, 2020. The first cohort to investigate antibiotic exposure during pregnancy (pregnancy analysis) included all children except for (1) those whose mothers were younger than 15 or older than 50 years, (2) those with chromosomal anomalies, (3) those whose mothers had taken antibiotics one month before but not during pregnancy to mitigate exposure misclassification, and (4) those whose mothers had no infection diagnosis during pregnancy. The second cohort to analyze antibiotic exposure in infancy and early childhood (infancy analysis) consisted of all children excluding (1) those who died within the first six months of life, (2) those with chromosomal anomalies, (3) those diagnosed with study outcomes within the first six months, and 4) those not diagnosed with an infection in the first six months of life (Fig 1).

### Antibiotics exposure

Exposure was defined as one or more prescriptions of systemic antibiotic, using *Anatomical Therapeutic Chemical classification system code*, J01 (S2 Table). Since systemic antibiotics in South Korea can only be obtained with a physician’s prescription, all instances of antibiotic use were included and exposure misclassification from the over-the-counter was unlikely. In the pregnancy analysis, the antibiotic exposed group consisted of children whose mothers at least one antibiotic prescription during pregnancy. The non-exposed group included children whose mothers were not prescribed antibiotics from 30 days before last menstrual period (LMP) until the end of pregnancy. For the infancy analysis, the exposed group comprised children who received antibiotics at least once within the first six months of life. The non-exposed group consisted of children who did not receive any antibiotic prescriptions during the same timeframe.

### Outcomes

The outcomes of interest included 6 autoimmune diseases including type 1 diabetes, juvenile idiopathic arthritis, ulcerative colitis, Crohn’s disease, systemic lupus erythematosus, and Hashimoto’s thyroiditis. All autoimmune disease events were required to have at least one inpatient or at least two outpatient diagnoses within one year for the improving specificity of the outcome (S2 Table). For the pregnancy analysis, children were followed from the day they were born until the occurrence of the outcome, death, or December 31, 2021. In the infancy analysis, children were followed from 180 days after birth until the occurrence of the outcome, death, or December 31, 2021.

### Sociodemographic, clinical, and obstetric characteristics

For the analyses of both pregnancy and infancy, we assessed a broad range of potential confounders: (1) maternal demographic characteristics (e.g., maternal age, insurance type, and income level), (2) clinical characteristics such as cause of infections (e.g., respiratory infection, urinary infection), proxies for severity of infection (e.g., infection-related number of outpatient visits, distinct diagnoses), maternal comorbid conditions (e.g., asthma, migraine), maternal medication conditions (e.g., systemic corticosteroids, chronic obstructive pulmonary disease drugs), measures of healthcare utilization (e.g., number of out- or in-patient visits), and (3) obstetric characteristics including nulliparity, multiple gestations, obstetric comorbidity index [27]. For infancy analysis, we additionally considered sex of child, maternal exposure to antibiotics, preterm birth, cesarean section, birth weight, and type of feeding. Maternal sociodemographic characteristics were assessed at the time of delivery. Clinical characteristics were examined from six months prior to pregnancy up until the day before delivery, while causes of infections and proxies for severity of infection were measured during pregnancy or during early infancy, respectively. Lastly, measures of healthcare utilization were assessed during the 6 months prior to pregnancy but not during pregnancy. Maternal lifestyle-related factors, including smoking habits and body mass index (BMI) status were also described based on data from the national health screening program conducted before the LMP.

### IPTW and sibling-matched analyses

We applied stabilized IPTW as our primary analytic approach to account for potential confounding. Although we initially planned to perform 1:1 propensity score matching using a greedy nearest neighbor algorithm without replacement, we revised our analytic strategy based on reviewer feedback and adopted IPTW to enhance statistical efficiency and generalizability, particularly given the comparable sizes of the antibiotics-exposed and unexposed groups. IPTW enabled the inclusion of a larger proportion of eligible subjects by assigning weights based on the probability of treatment, thereby reducing data loss while achieving covariate balance. We estimated PS, defined as the likelihood of antibiotic exposure in this study, through a logistic regression model that utilized all pre-defined covariates as independent variables. To prevent potential bias from extreme weights and to enhance exchangeability between two groups, we first performed 1% trimming within the overlapping region [28], followed by the application of stabilized IPTW. We assessed the balance of covariates between the exposed and unexposed groups using the standardized mean difference (SMD), considering values under 0.1 as indicative of balance [29,30]. We measured the incidence of outcomes per 100,000 person-years and calculated absolute rate differences with corresponding 95% confidence intervals (CIs) using Poisson regression. Hazard ratios (HRs) with 95% CIs were estimated using Cox proportional hazard regression models with robust standard errors to account for multiple pregnancies per mother.

As part of the main analysis, we also performed sibling-matched analyses restricted to children with siblings, including only sibling pairs who were discordant in exposure status, to account for potential unmeasured confounding by familial and maternal genetic factors. This approach inherently adjusts for time-invariant family-related characteristics. IRs and HRs for each outcome were estimated. HRs were derived using a stratified Cox proportional hazard regression model, with stratification by mother to account for familial clustering. The unit for all analyses was per child. Data management and analysis were conducted using SAS Enterprise Guide, version 7.1 (SAS Institute).

### Subgroup and sensitivity analyses

Several subgroup analyses were conducted. First, we conducted a subgroup analysis according to broad- and narrow-spectrum antibiotics to evaluate whether the risk differed by antibiotic spectrum. Second, we analyzed the risk of study outcomes by subclasses among antibiotics which are frequently prescribed within database during pregnancy (cephalosporins, imidazoles, macrolides, penicillins) or infancy (cephalosporins, macrolides, penicillins), given that different effects were reported according to each antibiotic class. Third, we evaluated the cumulative duration-response relationship between antibiotic exposure and autoimmune disease. In this analysis, we identified the distribution of cumulative duration within each cohort and categorized them based on quartiles (<4, 4–7, 8–12, 13+ days for the pregnancy analysis and <8, 8–16, 17–28, +29 days for the infancy analysis). Fourth, considering the differential incidence of certain autoimmune diseases based on sex, we conducted subgroup analyses by the sex of the infant. Lastly, we examined whether the risk of autoimmune disease varied according to specific timing of exposure, delineated as the 1st, 2nd, and 3rd trimester for the pregnancy analysis, and 0 to <2, 2 to <4, and 4 to <6 months of age after birth for the infancy analysis.

Additionally, sensitivity analyses were conducted to examine the robustness of the main analysis. First, we modified the definition of exposure to two or more prescriptions of systemic antibiotics within the same assessment window, to minimize exposure misclassification. Second, given the stronger genetic predisposition for autoimmune diseases among twins, their inclusion may lead to overestimation of associations due to unmeasured confounding. To address this, we conducted a sensitivity analysis restricted to singleton births [31]. Third, we limited the study population to children who were breastfed to address the residual confounding from not breastfeeding, given that breastfeeding plays a significant role in the formation of immunity [32]. Lastly, as the presence of maternal autoimmune diseases are critical risk factor for the development of such diseases in offspring, we excluded the study population to children whose mothers had diagnoses with autoimmune diseases [33].

## Results

### Baseline characteristics

For the pregnancy cohort, we identified 2,703,090 eligible children, among whom 1,516,574 (56.1%) were exposed to antibiotics in utero (Fig 1). Although restricted to children whose mothers were diagnosed with infection during pregnancy, proportion of cause of infection and proxies for the severity of infection (e.g., number of hospital visits with infection) were higher in the exposed group before PS weighting. Also, antibiotic exposure was more common among those with comorbidities of asthma and medication use (e.g., acetaminophen, NSAIDs) (Table 1). For the infancy cohort, among 3,347,049 eligible children with infections, 1,925,585 (57.5%) were exposed to antibiotics during early infancy. Before weighting, antibiotic-exposed infants were more likely to be male, have more causes of infection, and have mothers who were exposed to antibiotics during pregnancy (44.8% versus 39.4%) (Table 2).

**Table 1:** Baseline characteristics of cohort for pregnancy analysis before and after inverse probability of treatment weighting and sibling matching.

Characteristics	Before weighting			After weighting*			After sibling matching		
Maternal characteristics	Exposed	Unexposed	aSMD	Exposed	Unexposed	aSMD	Exposed	Unexposed	aSMD
	(*n* = 1,516,574)	(*n* = 1,186,516)		(*n* = 1,336,754)	(*n* = 1,041,825)		(*n* = 263,401)	(*n* = 257,313)	
**Demographic characteristics**									
Age at delivery, mean (SD)	31.9 (4.3)	31.9 (4.1)	0.01	31.9 (4.3)	31.9 (4.2)	0.00	31.6 (4.1)	31.1 (4.0)	**0.13**
Medical aid beneficiary, *n* (%)	12,133 (0.8)	5,933 (0.5)	0.04	8,021 (0.6)	6,251 (0.6)	0.00	1,580 (0.6)	1,287 (0.5)	0.01
Income level, quartile, *n* (%)			0.02			0.00			0.03
1st (most deprived)	308,099 (20.3)	227,923 (19.2)		263,946 (19.7)	205,489 (19.7)		53,774 (20.4)	53,028 (20.6)	
2nd	369,321 (24.4)	287,195 (24.2)		324,713 (24.3)	252,898 (24.3)		61,471 (23.3)	65,169 (25.3)	
3rd	521,343 (34.4)	416,813 (35.1)		464,349 (34.7)	362,304 (34.8)		91,490 (34.7)	88,214 (34.3)	
4th (most affluent)	317,811 (21.0)	254,585 (21.5)		283,746 (21.2)	221,134 (21.2)		56,666 (21.5)	50,902 (19.8)	
Metropolitan region, *n* (%)	1,049,774 (69.2)	858,641 (72.4)	0.07	945,200 (70.7)	736,930 (70.7)	0.00	182,183 (69.2)	179,693 (69.8)	0.02
Year of delivery, *n* (%)			0.02			0.00			0.08
2009–2011	386,490 (25.5)	310,746 (26.2)		348,719 (26.1)	271,798 (26.1)		48,465 (18.4)	59,785 (23.2)	
2012–2014	432,579 (28.5)	347,113 (29.3)		385,060 (28.8)	299,327 (28.7)		85,054 (32.3)	85,589 (33.3)	
2015–2017	389,363 (25.7)	320,303 (27.0)		350,061 (26.2)	272,863 (26.2)		79,231 (30.1)	77,380 (30.1)	
2018–2020	308,142 (20.3)	208,354 (17.6)		252,915 (18.9)	197,837 (19.0)		50,651 (19.2)	34,559 (13.4)	
**Cause of infections, *n* (%)**									
Respiratory infection	1,000,690 (66.0)	485,819 (40.9)	**0.52**	731,537 (54.7)	570,037 (54.7)	0.00	184,031 (69.9)	117,471 (45.7)	**0.51**
Genitourinary infection	998,941 (65.9)	781,668 (65.9)	0.00	862,486 (64.5)	672,384 (64.5)	0.00	170,846 (64.9)	167,221 (65.0)	0.00
Gastrointestinal infection	99,498 (6.6)	48,126 (4.1)	**0.11**	67,576 (5.1)	51,869 (5.0)	0.00	17,317 (6.6)	12,140 (4.7)	0.08
Sexually transmitted infection	103,902 (6.9)	38,555 (3.2)	**0.17**	60,237 (4.5)	48,004 (4.6)	0.01	16,993 (6.5)	8,481 (3.3)	**0.15**
Skin infection	224,500 (14.8)	108,943 (9.2)	**0.17**	151,700 (11.3)	118,410 (11.4)	0.00	39,384 (15.0)	25,394 (9.9)	**0.16**
Other infections	200,911 (13.2)	140,228 (11.8)	0.04	163,581 (12.2)	124,708 (12.0)	0.01	36,680 (13.9)	32,454 (12.6)	0.04
**Infection-related healthcare utilization**									
No. of outpatient visits, *n* (%)			**0.51**			0.01			**0.36**
0–1	9,925 (0.7)	3,171 (0.3)		6,871 (0.5)	5,493 (0.5)		1,336 (0.5)	607 (0.2)	
2	301,431 (19.9)	510,460 (43.0)		405,066 (30.3)	316,485 (30.4)		47,470 (18.0)	100,318 (39.0)	
3–4	728,046 (48.0)	545,231 (46.0)		665,907 (49.8)	521,129 (50.0)		126,886 (48.2)	124,707 (48.5)	
5+	477,172 (31.5)	127,654 (10.8)		258,910 (19.4)	198,718 (19.1)		87,709 (33.3)	31,681 (12.3)	
No. of distinct infection diagnoses, *n* (%)			**0.61**			0.00			**0.42**
0–1	386,870 (25.5)	639,565 (53.9)		524,590 (39.2)	409,896 (39.3)		60,459 (23.0)	126,121 (49.0)	
2–3	737,487 (48.6)	484,790 (40.9)		641,538 (48)	499,466 (47.9)		128,870 (48.9)	113,740 (44.2)	
4+	392,217 (25.9)	62,161 (5.2)		170,626 (12.8)	132,463 (12.7)		74,072 (28.1)	17,452 (6.8)	
ER visits with infection, *n* (%)	53,722 (3.5)	17,949 (1.6)	**0.12**	29,041 (2.2)	22,141 (2.1)	0.00	8,907 (3.4)	4,344 (1.8)	0.10
**Comorbid conditions, *n* (%)**									
Asthma	79,487 (5.2)	33,026 (2.8)	**0.13**	49,094 (3.7)	38,224 (3.7)	0.00	13,605 (5.2)	8,265 (3.2)	0.10
Chronic hypertension	40,177 (2.6)	22,425 (1.9)	0.05	29,635 (2.2)	23,326 (2.2)	0.00	5,109 (1.9)	4,363 (1.7)	0.02
Depression/mood disorder	21,554 (1.4)	11,070 (0.9)	0.05	14,778 (1.1)	11,513 (1.1)	0.00	3,008 (1.1)	2,588 (1.0)	0.01
Endometriosis	13,564 (0.9)	8,913 (0.8)	0.02	10,933 (0.8)	8,516 (0.8)	0.00	1,500 (0.6)	1,516 (0.6)	0.00
Polycystic ovarian syndrome	22,081 (1.5)	12,972 (1.1)	0.03	16,674 (1.2)	13,154 (1.3)	0.00	2,463 (0.9)	2,531 (1.0)	0.01
Renal disease	8,484 (0.6)	4,764 (0.4)	0.02	6,240 (0.5)	4,863 (0.5)	0.00	1,178 (0.4)	962 (0.4)	0.01
Rheumatic disease	7,771 (0.5)	4,857 (0.4)	0.02	6,102 (0.5)	4,730 (0.5)	0.00	1,131 (0.4)	996 (0.4)	0.01
Migraine	55,493 (3.7)	29,240 (2.5)	0.07	40,275 (3.0)	31,270 (3.0)	0.00	8,688 (3.3)	7,200 (2.8)	0.03
GI disorder	91,266 (6.0)	51,799 (4.4)	0.08	67,825 (5.1)	52,817 (5.1)	0.00	14,681 (5.6)	12,676 (4.9)	0.03
Anemia	162,918 (10.7)	117,368 (9.9)	0.03	137,452 (10.3)	107,496 (10.3)	0.00	27,285 (10.4)	25,793 (10.0)	0.01
**Medication conditions, *n* (%)**									
Acetaminophen	1,157,947 (76.4)	605,965 (51.1)	**0.55**	874,136 (65.4)	680,111 (65.3)	0.00	205,928 (78.2)	147,475 (57.3)	**0.46**
Antiepileptic drugs	15,704 (1.0)	8,430 (0.7)	0.04	11,274 (0.8)	8,886 (0.9)	0.00	2,278 (0.9)	1947 (0.8)	0.01
Antidepressants	35,530 (2.3)	18,051 (1.5)	0.06	24,344 (1.8)	19,002 (1.8)	0.00	4,968 (1.9)	4,237 (1.6)	0.02
Antihypertensives	86,726 (5.7)	32,594 (2.7)	**0.15**	50,903 (3.8)	40,414 (3.9)	0.00	11,619 (4.4)	7,184 (2.8)	0.09
Benzodiazepines	186,615 (12.3)	100,052 (8.4)	**0.13**	134,317 (10.0)	104,973 (10.1)	0.00	26,531 (10.1)	23,016 (8.9)	0.04
NSAIDs	1,066,028 (70.3)	631,526 (53.2)	**0.36**	834,777 (62.4)	648,873 (62.3)	0.00	184,553 (70.1)	149,386 (58.1)	**0.25**
Fertility drugs	125,540 (8.3)	76,649 (6.5)	0.07	98,081 (7.3)	76,849 (7.4)	0.00	14,702 (5.6)	14,632 (5.7)	0.01
Thyroid medications	80,272 (5.3)	56,908 (4.8)	0.02	67,209 (5.0)	52,976 (5.1)	0.00	13,028 (4.9)	11,287 (4.4)	0.03
Lipid-lowering drugs	13,759 (0.9)	6,301 (0.5)	0.05	8,980 (0.7)	7,044 (0.7)	0.00	2,403 (0.9)	1,446 (0.6)	0.04
Antiacids	952,630 (62.8)	549,106 (46.3)	**0.34**	732,155 (54.8)	569,158 (54.6)	0.00	164,678 (62.5)	130,368 (50.7)	**0.24**
Systemic corticosteroids	735,414 (48.5)	421,325 (35.5)	**0.27**	553,365 (41.4)	430,428 (41.3)	0.00	125,808 (47.8)	100,154 (38.9)	**0.18**
Triptans	5,382 (0.4)	2,719 (0.2)	0.02	3,742 (0.3)	2,923 (0.3)	0.00	826 (0.3)	690 (0.3)	0.01
Antiemetics	337,711 (22.3)	182,398 (15.4)	**0.18**	245,261 (18.3)	191,379 (18.4)	0.00	53,862 (20.4)	44,738 (17.4)	0.08
COPD drugs	342,549 (22.6)	153,062 (12.9)	**0.26**	224,858 (16.8)	174,194 (16.7)	0.00	63,594 (24.1)	38,789 (15.1)	**0.23**
Immunmodulator	19,430 (1.3)	10,902 (0.9)	0.04	14,035 (1.0)	10,978 (1.1)	0.00	2,899 (1.1)	2,256 (0.9)	0.02
**Obstetric conditions, *n* (%)**									
Nulliparity	718,958 (47.4)	638,451 (53.8)	**0.13**	678,119 (50.7)	531,405 (51.0)	0.01	86,752 (32.9)	127,169 (49.4)	**0.34**
Multiple gestations	58,401 (3.9)	27,627 (2.3)	0.09	40,550 (3)	31,947 (3.1)	0.00	4,723 (1.8)	2,665 (1.0)	0.06
Obstetric comorbidity index, *n* (%)			**0.13**			0.00			**0.17**
0	832,612 (54.9)	721,864 (60.8)		774,440 (57.9)	602,326 (57.8)		151,552 (57.5)	168,198 (65.4)	
1	411,404 (27.1)	305,132 (25.7)		353,955 (26.5)	276,043 (26.5)		73,562 (27.9)	61,861 (24.0)	
2+	272,558 (18.0)	159,520 (13.4)		208,358 (15.6)	163,456 (15.7)		38,287 (14.5)	27,254 (10.6)	
**Healthcare utilization**									
No. of outpatient visits, *n* (%)			**0.24**			0.00			0.04
0	136,399 (9.0)	172,832 (14.6)		156,403 (11.7)	122,157 (11.7)		22,948 (8.7)	28,991 (11.3)	
1–2	293,352 (19.3)	298,982 (25.2)		299,486 (22.4)	233,353 (22.4)		54,040 (20.5)	60,600 (23.6)	
3–8	665,023 (43.9)	501,255 (42.2)		589,560 (44.1)	459,686 (44.1)		120,061 (45.6)	115,319 (44.8)	
9+	421,800 (27.8)	213,447 (18.0)		291,306 (21.8)	226,628 (21.8)		66,352 (25.2)	52,403 (20.4)	
Inpatient hospitalizations, *n* (%)			0.07			0.00			0.02
0	1,400,489 (92.3)	1,118,924 (94.3)		1,249,551 (93.5)	973,710 (93.5)		243,277 (92.4)	239,510 (93.1)	
1	98,437 (6.5)	59,661 (5.0)		75,931 (5.7)	59,419 (5.7)		17,446 (6.6)	15,593 (6.1)	
2+	17,648 (1.2)	7,931 (0.7)		11,272 (0.8)	8,696 (0.8)		2,678 (1)	2,210 (0.9)	
ER visits, *n* (%)			0.08			0.00			0.02
0	1,397,799 (92.2)	1,118,060 (94.2)		1,247,779 (93.3)	972,646 (93.4)		244,357 (92.8)	239,948 (93.3)	
1	97,768 (6.4)	58,447 (4.9)		75,074 (5.6)	58,362 (5.6)		15,949 (6.1)	14,667 (5.7)	
2+	21,007 (1.4)	10,009 (0.8)		13,901 (1.0)	10,817 (1.0)		3,095 (1.2)	2,698 (1.0)	
**Smoking habits, *n* (%)**			0.03			0.00			0.02
Ever	32,656 (2.2)	21,307 (1.8)		26,235 (2.0)	20,660 (2.0)		5,271 (2.0)	5,177 (2.0)	
Never	642,848 (42.4)	523,189 (44.1)		577,370 (43.2)	450,753 (43.3)		117,172 (44.5)	112,584 (43.8)	
Missing	841,070 (55.5)	642,020 (54.1)		733,149 (54.8)	570,411 (54.8)		140,958 (53.5)	139,552 (54.2)	
**BMI status, *n* (%)**			0.03			0.00			0.01
<18.5 (Underweight)	82,960 (5.5)	70,499 (5.9)		75,881 (5.7)	59,116 (5.7)		15,911 (6)	15,630 (6.1)	
18.5–22.9 (Normal)	486,014 (32)	401,920 (33.9)		440,432 (32.9)	344,072 (33.0)		88,989 (33.8)	86,499 (33.6)	
23.0–24.9 (Overweight)	84,539 (5.6)	59,117 (5.0)		70,695 (5.3)	55,179 (5.3)		14,373 (5.5)	12,939 (5.0)	
≥25.0 (Obese)	20,623 (1.4)	11,815 (1.0)		15,384 (1.2)	12,069 (1.2)		3,004 (1.1)	2,558 (1.0)	
Missing	842,438 (55.5)	643,165 (54.2)		734,363 (54.9)	571,389 (54.8)		141,124 (53.6)	139,687 (54.3)	

Abbreviations: aSMD, absolute standardized mean difference; COPD, chronic obstructive pulmonary disease; GI, gastrointestinal; SD, standard deviation; ER, emergency room; NSAIDs, non-steroidal anti-inflammatory drugs.

*Weighted number and percentages.

**Table 2:** Baseline characteristics of cohort for early infancy analysis before and after inverse probability of treatment weighting and sibling matching.

Characteristics	Before weighting			After weighting*			After sibling matching		
	Exposed	Unexposed	aSMD	Exposed	Unexposed	aSMD	Exposed	Unexposed	aSMD
	(*n* = 1,925,585)	(*n* = 1,421,464)		(*n* = 1,403,667)	(*n* = 1,277,387)		(*n* = 421,725)	(*n* = 401,729)	
**Maternal characteristics**									
**Demographic characteristics**									
Age at delivery, mean (SD)	31.9 (4.1)	32 (4.3)	0.01	31.9 (4.2)	31.9 (4.2)	0.00	31.9 (3.9)	31 (4.0)	**0.23**
Medical aid beneficiary, *n* (%)	13,657 (0.7)	7,029 (0.5)	0.03	8,388 (0.6)	7,598 (0.6)	0.00	2,171 (0.5)	2,173 (0.5)	0.00
Income level, quartile, *n* (%)			0.02			0.00			0.06
1st (Most deprived)	386,607 (20.1)	272,138 (19.1)		277,850 (19.8)	252,932 (19.8)		81,958 (19.4)	83,473 (20.8)	
2nd	460,206 (23.9)	345,183 (24.3)		341,362 (24.3)	310,372 (24.3)		92,546 (21.9)	104,633 (26.0)	
3rd	674,502 (35.0)	491,749 (34.6)		487,611 (34.7)	443,938 (34.8)		151,602 (35.9)	136,209 (33.9)	
4th (most affluent)	1,397,325 (20.6)	310,692 (21.9)		296,844 (21.1)	270,145 (21.1)		94,391 (22.4)	76,968 (19.2)	
Metropolitan region, *n* (%)	1,281,199 (66.5)	1,029,260 (72.4)	**0.13**	983,774 (70.1)	897,373 (70.3)	0.00	283,248 (67.2)	275,251 (68.5)	0.03
Year of delivery, *n* (%)			**0.12**			0.00			**0.21**
2009–2011	619,831 (32.2)	337,919 (23.8)		393,359 (28.0)	352,366 (27.6)		70,060 (16.6)	108,563 (27.0)	
2012–2014	578,122 (30.0)	434,901 (30.6)		438,425 (31.2)	402,710 (31.5)		135,768 (32.2)	147,939 (36.8)	
2015–2018	587,841 (30.5)	470,554 (33.1)		445,328 (31.7)	405,494 (31.7)		176,804 (41.9)	119,708 (29.8)	
2019–2020	139,791 (7.3)	178,090 (12.5)		126,555 (9.0)	116,818 (9.1)		39,093 (9.3)	25,519 (6.4)	
**Comorbid conditions, *n* (%)**									
Asthma	83,384 (4.3)	44,304 (3.1)	0.06	50,496 (3.6)	45,838 (3.6)	0.00	18,047 (4.3)	13,345 (3.3)	0.05
Chronic hypertension	42,086 (2.2)	29,449 (2.1)	0.01	30,911 (2.2)	28,546 (2.2)	0.00	6,964 (1.7)	7,024 (1.7)	0.01
Depression/mood disorder	21,128 (1.1)	14,830 (1.0)	0.01	14,887 (1.1)	13,681 (1.1)	0.00	4,031 (1.0)	3,715 (0.9)	0.00
Endometriosis	13,515 (0.7)	13,570 (1.0)	0.03	11,635 (0.8)	10,868 (0.9)	0.00	2082 (0.5)	2,855 (0.7)	0.03
Polycystic ovarian syndrome	20,855 (1.1)	20,606 (1.4)	0.03	17,467 (1.2)	16,245 (1.3)	0.00	3,446 (0.8)	4,612 (1.1)	0.03
Renal disease	9,067 (0.5)	6,181 (0.4)	0.01	6,500 (0.5)	5,825 (0.5)	0.00	1,517 (0.4)	1,628 (0.4)	0.01
Rheumatic disease	8,897 (0.5)	6,066 (0.4)	0.01	6,228 (0.4)	5,800 (0.5)	0.00	1,627 (0.4)	1,535 (0.4)	0.00
Migraine	60,222 (3.1)	37,069 (2.6)	0.03	39,313 (2.8)	35,694 (2.8)	0.00	11,988 (2.8)	10,934 (2.7)	0.01
GI disorder	97,239 (5.0)	68,266 (4.8)	0.01	68,401 (4.9)	62,324 (4.9)	0.00	19,731 (4.7)	20,735 (5.2)	0.02
Anemia	202,591 (10.5)	147,002 (10.3)	0.01	147,049 (10.5)	134,133 (10.5)	0.00	42,825 (10.2)	42,560 (10.6)	0.01
**Medication conditions, *n* (%)**									
Acetaminophen	1,212,809 (63.0)	777,665 (54.7)	**0.17**	816,734 (58.2)	742,537 (58.1)	0.00	278,140 (66.0)	229,737 (57.2)	**0.18**
Antiepileptic drugs	16,506 (0.9)	11,071 (0.8)	0.01	11,503 (0.8)	10,538 (0.8)	0.00	3,194 (0.8)	2,994 (0.7)	0.00
Antidepressants	35,734 (1.9)	24,022 (1.7)	0.01	24,620 (1.8)	22,519 (1.8)	0.00	6,593 (1.6)	6,266 (1.6)	0.00
Antihypertensives	78,316 (4.1)	53,606 (3.8)	0.02	55,821 (4.0)	51,406 (4.0)	0.00	14,972 (3.6)	12,897 (3.2)	0.02
Benzodiazepines	200,114 (10.4)	136,372 (9.6)	0.03	139,204 (9.9)	127,103 (10.0)	0.00	35,993 (8.5)	37,795 (9.4)	0.03
NSAIDs	1,208,289 (62.7)	815,500 (57.4)	**0.11**	835,663 (59.5)	760,328 (59.5)	0.00	266,333 (63.2)	237,622 (59.1)	0.08
Fertility drugs	131,300 (6.8)	115,774 (8.1)	0.05	104,816 (7.5)	96,195 (7.5)	0.00	20,537 (4.9)	27,073 (6.7)	0.08
Thyroid medications	85,659 (4.4)	74,524 (5.2)	0.04	66,278 (4.7)	61,094 (4.8)	0.00	20,226 (4.8)	16,429 (4.1)	0.03
Lipid-lowering drugs	12,952 (0.7)	8,923 (0.6)	0.01	9,133 (0.7)	8,401 (0.7)	0.00	3,448 (0.8)	2032 (0.5)	0.04
Antiacids	1,032,773 (53.6)	714,717 (50.3)	0.07	721,258 (51.4)	657,227 (51.5)	0.00	232,348 (55.1)	205,000 (51.0)	0.08
Systemic corticosteroids	790,106 (41.0)	545,720 (38.4)	0.05	553,494 (39.4)	505,545 (39.6)	0.00	176,038 (41.7)	156,807 (39.0)	0.06
Triptans	5,424 (0.3)	3,610 (0.3)	0.01	3,665 (0.3)	3,291 (0.3)	0.00	1,117 (0.3)	968 (0.2)	0.01
Antiemetics	357,933 (18.6)	242,379 (17.1)	0.04	247,981 (17.7)	226,546 (17.7)	0.00	73,469 (17.4)	72,552 (18.1)	0.02
COPD drugs	355,650 (18.5)	201,074 (14.1)	**0.12**	222,234 (15.8)	202,104 (15.8)	0.00	85,845 (20.4)	60,456 (15.0)	**0.14**
Immunmodulator	17,838 (0.9)	16,013 (1.1)	0.02	14,330 (1.0)	13,181 (1.0)	0.00	3,466 (0.8)	4,079 (1.0)	0.02
**Obstetric conditions, *n* (%)**									
Nulliparity	711,787 (37.0)	898,751 (63.2)	**0.54**	724,841 (51.6)	669,601 (52.4)	0.02	90,549 (21.5)	253,466 (63.1)	**0.93**
Multiple gestations	63,824 (3.3)	43,279 (3.0)	0.02	47,161 (3.4)	43,386 (3.4)	0.00	8,629 (2.0)	5,339 (1.3)	0.06
Obstetric comorbidity index, *n* (%)			0.07			0.00			**0.21**
0	1,141,070 (59.3)	897,962 (63.2)		860,417 (61.3)	782,896 (61.3)		248,687 (59.0)	282,459 (70.3)	
1	493,623 (25.6)	327,987 (23.1)		336,589 (24.0)	305,877 (23.9)		114,939 (27.3)	80,917 (20.1)	
2+	290,892 (15.1)	195,515 (13.8)		206,661 (14.7)	188,614 (14.8)		58,099 (13.8)	38,353 (9.5)	
**Healthcare utilization**									
No. of outpatient visits, *n* (%)			0.03			0.00			0.02
0	368,851 (19.2)	294,443 (20.7)		284,758 (20.3)	258,711 (20.3)		76,985 (18.3)	78,544 (19.6)	
1	207,639 (10.8)	165,296 (11.6)		159,319 (11.4)	143,963 (11.3)		46,800 (11.1)	46,200 (11.5)	
2-7	882,681 (45.8)	642,939 (45.2)		638,465 (45.5)	580,790 (45.5)		198,215 (47.0)	187,849 (46.8)	
8+	466,414 (24.2)	318,786 (22.4)		321,125 (22.9)	293,923 (23.0)		99,725 (23.6)	89,136 (22.2)	
Inpatient hospitalizations, *n* (%)			0.04			0.00			0.05
0	1,802,512 (93.6)	1,343,499 (94.5)		1,322,263 (94.2)	1,203,092 (94.2)		392,396 (93)	378,785 (94.3)	
1	106,172 (5.5)	67,863 (4.8)		70,682 (5.0)	64,395 (5.0)		25,668 (6.1)	19,960 (5.0)	
2+	16,901 (0.9)	10,102 (0.7)		10,722 (0.8)	9,900 (0.8)		3,661 (0.9)	2,984 (0.7)	
ER visits, *n* (%)			0.01			0.00			0.01
0	1,809,788 (94)	1,340,505 (94.3)		1,322,567 (94.2)	1,203,237 (94.2)		396,621 (94)	377,776 (94)	
1	97,147 (5.0)	68,306 (4.8)		68,308 (4.9)	62,444 (4.9)		21,218 (5.0)	20,232 (5.0)	
2+	18,650 (1.0)	12,653 (0.9)		12,792 (0.9)	11,706 (0.9)		3,886 (0.9)	3,721 (0.9)	
**Smoking habits, *n* (%)**			**0.16**			0.01			0.01
Ever	32,820 (1.7)	27,738 (2.0)		25,931 (1.8)	24,096 (1.9)		7,533 (1.8)	7,395 (1.8)	
Never	738,598 (38.4)	655,941 (46.1)		590,051 (42.0)	539,791 (42.3)		187,950 (44.6)	176,602 (44.0)	
Missing	1,154,167 (59.9)	737,785 (51.9)		787,685 (56.1)	713,500 (55.9)		226,242 (53.6)	217,732 (54.2)	
**BMI status, *n* (%)**			**0.15**			0.01			0.01
<18.5 (Underweight)	90,756 (4.7)	91,582 (6.4)		76,841 (5.5)	70,323 (5.5)		24,937 (5.9)	24,536 (6.1)	
18.5–22.9 (Normal)	559,234 (29.0)	502,698 (35.4)		450,061 (32.1)	412,034 (32.3)		143,728 (34.1)	136,193 (33.9)	
23.0–24.9 (Overweight)	97,992 (5.1)	72,849 (5.1)		72,334 (5.2)	66,120 (5.2)		22,100 (5.2)	19,301 (4.8)	
≥25.0 (Obese)	22,515 (1.2)	15,119 (1.1)		15,868 (1.1)	14,621 (1.1)		4,498 (1.1)	3,853 (1.0)	
Missing	1,155,088 (60.0)	739,216 (52.0)		788,562 (56.2)	714,289 (55.9)		226,462 (53.7)	217,846 (54.2)	
**Infant characteristics**									
**Sex**			**0.11**			0.00			**0.13**
Male	1,049,254 (54.7)	695,364 (49.0)		723,164 (51.5)	658,895 (51.6)		227,268 (54.0)	191,054 (47.6)	
Female	869,386 (45.3)	724,398 (51.0)		680,502 (48.5)	618,492 (48.4)		193,229 (46.0)	210,229 (52.4)	
**Maternal antibiotic exposure, *n* (%)**	862,406 (44.8)	559,456 (39.4)	**0.11**	585,544 (41.7)	532,969 (41.7)	0.00	190,695 (45.2)	159,134 (39.6)	**0.23**
Preterm birth	258,939 (13.4)	190,190 (13.4)	0.00	196,864 (14.0)	182,371 (14.3)	0.01	50,587 (12.0)	53,051 (13.2)	0.04
Cesarean birth	802,385 (41.7)	596,091 (41.9)	0.01	586,765 (41.8)	535,596 (41.9)	0.00	158,749 (37.6)	145,451 (36.2)	0.03
**Birth weight, *n* (%)** †			0.02			0.07			0.06
+2.5 kg	1,713,415 (89.0)	1,269,222 (89.3)		1,229,627 (87.6)	1,148,455 (89.9)		382,894 (90.8)	371,274 (92.4)	
<1.5 kg	13,166 (0.7)	2,697 (0.2)		12,680 (0.9)	2,623 (0.2)		1812 (0.4)	723 (0.2)	
1.5-2.5 kg	128,498 (6.7)	79,233 (5.6)		103,081 (7.3)	72,737 (5.7)		22,333 (5.3)	17,856 (4.4)	
Missing	70,506 (3.7)	70,312 (4.9)		58,278 (4.2)	53,573 (4.2)		14,686 (3.5)	11,876 (3.0)	
**Feeding, *n* (%)** †			0.06			0.02			0.09
Breast	406,888 (21.1)	333,750 (23.5)		299,127 (21.3)	309,868 (24.3)		99,908 (23.7)	113,922 (28.4)	
Formula	633,598 (32.9)	476,216 (33.5)		467,417 (33.3)	413,583 (32.4)		143,685 (34.1)	118,864 (29.6)	
Mixed	217,613 (11.3)	189,791 (13.4)		170,276 (12.1)	163,928 (12.8)		47,828 (11.3)	56,652 (14.1)	
Missing	660,541 (34.3)	420,005 (29.5)		466,847 (33.3)	390,009 (30.5)		129,076 (30.6)	111,845 (27.8)	
**Cause of infections, *n* (%)**									
Respiratory infection	1,867,527 (97.0)	1,273,774 (89.6)	**0.30**	1,330,314 (94.8)	1,209,074 (94.7)	0.01	412,814 (97.9)	368,860 (91.8)	**0.28**
Urinary infection	166,534 (8.6)	23,053 (1.6)	**0.32**	42,200 (3.0)	39,891 (3.1)	0.01	34,273 (8.1)	6,639 (1.7)	**0.30**
Skin infection	883,100 (45.9)	239,051 (16.8)	**0.66**	393,252 (28.0)	354,588 (27.8)	0.01	207,905 (49.3)	65,876 (16.4)	**0.75**
Other infections	516,057 (26.8)	318,024 (22.4)	**0.10**	304,359 (21.7)	280,688 (22.0)	0.01	111,106 (26.3)	87,629 (21.8)	**0.11**

Abbreviations: aSMD, standardized mean difference; COPD, chronic obstructive pulmonary disease; GI, gastrointestinal; SD, standard deviation; ER, emergency room; NSAIDs, non-steroidal anti-inflammatory drugs.

*Weighted number and percentages.

†Birth weight and feeding were not included in the PS calculation model nor outcome modeling and presented as baseline characteristics for comparison between antibiotics exposed and nonexposed group.

After IPTW, we identified 1,336,754 exposed and 1,041,825 unexposed for pregnancy analysis, and 1,403,667 exposed infants and 1,277,387 unexposed infants for the infancy analysis, and all characteristics were well-balanced between the groups (aSMD < 0.1) (Table 1 and 2).

### Exposure to antibiotics during pregnancy: IPTW and sibling-matched analyses

In pregnancy analysis, each group for respective outcome of interest was followed for a median duration of 7.6 years. The infection-restricted IPTW analyses showed null association between antibiotic exposure and autoimmune diseases in children, including type 1 diabetes (IPTW HR 1.14, 95% CI [0.96, 1.35]; *p*-value = 0.132), juvenile idiopathic arthritis (HR 1.02, 95% CI [0.85, 1.22]; *p*-value = 0.830), ulcerative colitis (HR 1.02, 95% CI [0.76, 1.37]; *p*-value = 0.895), Crohn’s disease (HR 1.16, 95% CI [0.98, 1.36]; *p*-value = 0.076), systemic lupus erythematosus (HR 0.70, 95% CI [0.49, 1.01]; *p*-value = 0.053), and Hashimoto’s thyroiditis (HR 1.06, 95% CI [0.91, 1.23]; *p*-value = 0.448).

**Fig 2:**
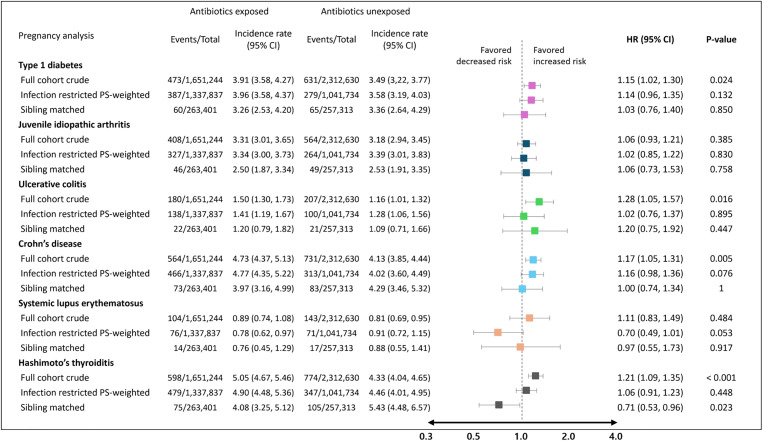
Risk of autoimmune disease associated with antibiotic exposure during pregnancy. Abbreviations: HR, hazard ratio; PS, propensity score. *Incidence rate of respective outcome of interest was calculated per 100,000 person-years. ^†^In the full cohort, crude analyses indicated a statistically significant increased risk of type 1 diabetes, ulcerative colitis, Crohn’s disease, and Hashimoto’s thyroiditis. However, when the analysis was restricted to individuals with infections and inverse probability of treatment weighting was applied, as well as in analyses restricted to exposure-discordant sibling pairs, all associations across the six autoimmune diseases were attenuated to null.

Similarly, in the infection-restricted sibling-matched analyses, 263,401 exposed and 257,313 unexposed siblings were identified, and no associations were observed between antibiotic exposure during pregnancy and all 6 autoimmune diseases (Fig 2).

### Exposure to antibiotics during pregnancy: Subgroup and sensitivity analyses

In the subgroup analysis by antibiotic spectrum within the pregnancy cohort, exposure to broad-spectrum antibiotics was associated with a modestly increased risk of Crohn’s disease (IPTW HR 1.19, 95% CI [1.00, 1.42]) (S3 Table). Elevated risks were also observed for Crohn’s disease in association with maternal exposure to cephalosporins (HR 1.29, 95% CI [1.07, 1.57]) and with exposure during the first and second trimesters (S4 and S7 Tables). No duration-dependent trends or effect modification by child sex were observed for any of the outcomes (S5 and S6 Tables). The results of the sensitivity analyses were consistent with the primary findings, except for Crohn’s disease, which showed an increased risk when exposure was redefined as two or more antibiotic prescriptions (S13 Table).

### Exposure to antibiotics during early infancy: IPTW and sibling-matched analyses

In infancy analysis, each outcome of interest was followed for a median duration of 7.4 years. In the infection-restricted IPTW analyses, we found no difference in incidence of autoimmune disease risk between exposed and non-exposed groups for type 1 diabetes (IPTW HR 1.05, 95% CI [0.88, 1.26]; *p*-value = 0.594) and juvenile idiopathic arthritis (HR 1.11, 95% CI [0.93, 1.33]; *p*-value = 0.253), ulcerative colitis (HR 0.95, 95% CI [0.67, 1.36], *p*-value = 0.776), Crohn’s disease (HR 1.07, 95% CI [0.91, 1.25]; *p*-value = 0.403), systemic lupus erythematosus (HR 1.46, 95% CI [0.95, 2.26]; *p*-value = 0.087), and Hashimoto’s thyroiditis (HR 1.14, 95% CI [0.97, 1.33]; *p*-value = 0.104).

**Fig 3:**
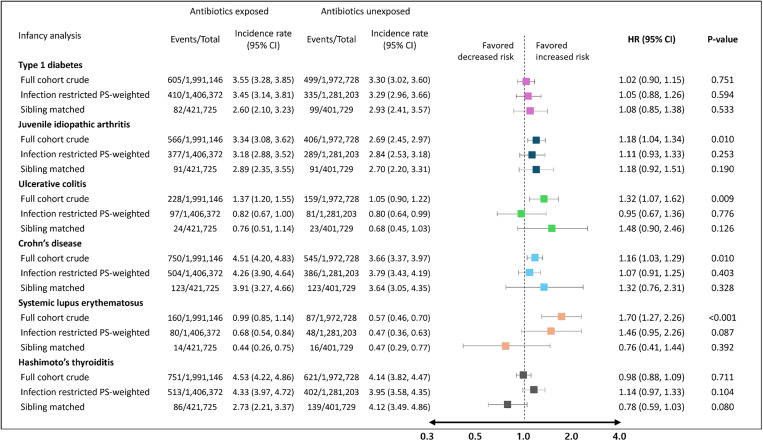
Risk of autoimmune disease associated with antibiotic exposure during early infancy. Abbreviations: HR, hazard ratio; CI, confidence interval; PS, propensity score. *Incidence rate of respective outcome of interest was calculated per 100,000 person-years. ^†^In the full cohort, crude analyses indicated a statistically significant increased risk of juvenile idiopathic arthritis, ulcerative colitis, Crohn’s disease, and systemic lupus erythematosus. However, when the analysis was restricted to individuals with infections and inverse probability of treatment weighting was applied, as well as in analyses restricted to exposure-discordant sibling pairs, all associations across the six autoimmune diseases were attenuated to null.

In the infection-restricted sibling-matched analyses, we included 421,725 exposed and 401,729 unexposed siblings for the infancy analysis. The sibling-matched HRs also consistently suggest no association between antibiotic exposure during early infancy and all 6 autoimmune diseases (Fig 3).

### Exposure to antibiotics early infancy: Subgroup and sensitivity analyses

In the IPTW analyses, no associations were observed by antibiotic spectrum, individual subclasses, or duration-dependent trends for any of the outcomes (S8, S9 and S10 Tables). However, the risk of Hashimoto’s thyroiditis was increased among males (IPTW HR 1.40, 95% CI [1.02, 1.90]) and following antibiotic exposure within two months after birth (HR 1.30, 95% CI [1.07, 1.58]) (S11 and S12 Tables). The findings from sensitivity analyses demonstrated substantial consistency with our primary results (S14 Table).

## Discussion

In this large-scale nationwide cohort study of over 4 million individuals using a mother–child linked database, we examined the association between antibiotic exposure during pregnancy or early infancy and the risk of autoimmune diseases in children. While our findings rule out a substantial increase in autoimmune disease risk, the study may have been underpowered to detect small but potentially meaningful associations, particularly within certain subgroups. Sensitivity analyses including redefining exposure as two or more prescriptions, restricting the cohort to singleton births or to children who were breastfed, and excluding those whose mothers had a diagnosis of autoimmune disease produced results consistent with the primary analysis.

Building upon our findings, it is important to place them in the context of existing literature on prenatal and early-life antibiotic exposure and autoimmune outcomes. Previous studies have explored the association between prenatal antibiotic exposure and the development of autoimmune diseases in offspring, with conflicting findings. A Swedish study reported an association between exposure to antibiotics during pregnancy and the development inflammatory bowel disease (IBD) (adjusted HR 1.93, 95% CI [1.06, 3.50]), but they failed to conduct sibling analysis, which could control for both genetic and environmental factors due to the small number of cases [16]. On the other hand, Norwegian register-based and Danish prospective cohort studies found no associations between prenatal antibiotics and juvenile idiopathic arthritis or type 1 diabetes [19,23]. In line with this previous literature, we found no association between prenatal or early-life exposure to antibiotics and the risk of any autoimmune diseases. These results are likely to be supported by the hypothesis that the fetal environment in utero is considered sterile, with microbial colonization commencing post-birth, and thus unlikely impacting children’s immune development [34,35]. However, due to the reduced precision in some outcomes, more research is needed in this area to provide a deeper understanding of the potential effects of antibiotics during pregnancy.

For the antibiotics during infancy period, a register-based study from Sweden showed an increased risk of type 1 diabetes following the use of antibiotics (adjusted HR 1.19, 95% CI [1.05, 1.36] and sibling-matched HR 1.36, 95% CI [0.99, 1.88]) [21], lacking consideration for various confounding variables such as maternal comorbidities or comedications. Also, a substantial number of studies found increased risk, revealing a dose-response relationship, especially for juvenile idiopathic arthritis and Crohn’s disease [15,18,36–39], which have often overlooked the risk factors like infection severity which could mislead to have dose-response relationship if not controlled sufficiently, and also genetic/environmental factors. At the same time, other studies found no evidence of an association between antibiotic use and type 1 diabetes or IBD [16,40–42]. Consistent with these findings, our study did not demonstrate associations or a clear dose-response relationship after rigorous control for more than 40 potential confounders, as well as the severity of infection and genetic/environmental factors through infection-restricted and sibling-matched analyses. Given the high prevalence of antibiotic prescriptions in current clinical settings, our study’s design minimizes biases, underscoring the necessity of considering confounding factors in understanding the association between early antibiotic use and autoimmune disease among children.

Infection is a well-established contributor to the development of autoimmune diseases, with several mechanisms proposed, including polyclonal lymphocyte activation, molecular mimicry, and heightened immunogenicity of organ-specific autoantigens resulting from infection-induced inflammation [43]. Of particular relevance, bacterial infections that are commonly treated with antibiotics can initiate immune responses via bacterial DNA, which functions as a pathogen-associated molecular pattern that activates Toll-like receptor 9 (TLR9) following endosomal translocation [44]. To reduce potential confounding by infection, we restricted our analysis to individuals with diagnoses of infections. This design allowed for a more precise assessment of the independent association between antibiotic exposure and autoimmune disease risk in children.

There is a biologically plausible mechanism by which antibiotics administered during pregnancy may influence fetal development. Antibiotics are metabolized by the maternal microbiota and may reach the developing fetus directly. Alternatively, maternal gut microbiome disruption caused by antibiotic use may influence immune shaping in the offspring by altering early immune system development, particularly T-cell differentiation and regulatory T-cell (Treg) balance, which are critical for immune tolerance [35,45,46]. Disruptions in these pathways may increase susceptibility to autoimmune responses later in life. Likewise, exposure to antibiotics during infancy can induce alterations in the gastrointestinal microbiota, leading to weakened gut mucosal barrier integrity [10,47]. This breakdown allows for an increased influx of antigens and heightened immune activation, influencing both innate and adaptive immune responses. Specifically, dysbiosis has been linked to altered Th17 cell activity and B cell maturation, which are implicated in autoimmune pathogenesis. These mechanisms underscore the potential role of early-life antibiotic exposure in shaping immune function and susceptibility to autoimmune diseases [8]. Despite the plausible mechanisms linking antibiotic exposure to the development of autoimmune diseases, we observed null findings after adjusting for multiple risk factors, including over 40 known confounders, and accounting for potential familial influences. By addressing these variables, we aimed to reduce potential bias and enhance the validity of our findings.

Interestingly, although the overall associations between prenatal or early-life antibiotic exposure and the risk of autoimmune diseases were largely null after rigorous confounding adjustment, subgroup analyses revealed elevated risks in specific populations or conditions. First, maternal cephalosporins use or antibiotics use during early to mid-pregnancy was associated with an increased risk of Crohn’s disease in offspring. Cephalosporins, as third- and fourth-generation β-lactams, can cause sustained disruption of the maternal gut microbiota, potentially interfering with fetal immune development and tolerance [48,49]. Given their widespread use in pregnancy, further pharmacologic and epidemiologic studies are warranted to assess long-term immunological consequences of the use of this antibiotic class. Also, during the first and second trimesters, the foundational architecture of the immune system is established, including thymic development, T-cell differentiation, and the formation of central immune tolerance [50,51]. Disruptions to maternal microbial signals during this immunologically sensitive window may alter immune programming in the fetus and potentially increase susceptibility to immune-mediated conditions later in life [51]. Second, antibiotic exposure in males or during the first two months of life was associated with a modestly increased risk of autoimmune thyroiditis. This sex-specific susceptibility is supported by known immunological differences between male and female infants, as male infants show delayed Treg development and reduced type I interferon responses [52,53]. Further, the early infancy period represents a critical window for immune maturation and microbial colonization, during which antibiotic-induced dysbiosis may have lasting effects [54]. These findings highlight the need for future research into the class-, sex-, and timing-specific effects of antibiotic exposure on immune programming and the risk of autoimmune diseases in children.

There are several limitations that must be taken into account when interpreting this study. First, due to the inherent limitations of observational research, which cannot fully eliminate the influence of unmeasured or unknown confounding factors, we implemented various strategies such as IPTW and sibling matching, while adjusting for multiple covariates and proxy variables related to pregnancy and early infancy (e.g., breastfeeding status, socioeconomic status, education, geographic disparities in healthcare utilization). However, we acknowledge that certain residual confounders, such as dietary factors, environmental exposures, and other lifestyle-related influences, are not explicitly recorded in the NHIS–NHID database. Second, while we cannot definitively rule out the possibility that immune-related diseases are caused by infection rather than antibiotic exposure, we addressed this concern by focusing our study population on those with infections and extensively adjusting for infection-related covariates. Third, there could be residual confounding due to maternal autoimmunity on children’s outcomes. To address this, we conducted sibling-matched analyses to account for shared genetic factors, including paternal autoimmunity, and performed sensitivity analyses excluding mothers with autoimmune diseases. Fourth, there is potential for exposure misclassification. However, in our sensitivity analysis, we defined antibiotic exposure as two or more prescriptions, and the results were consistent with those of the main analysis. Fifth, this study examines the association between antibiotic exposure during pregnancy and infancy on autoimmune disease risk, with a median follow-up of 7 years, and a maximum of 14 years. While this follow-up duration is generally sufficient to assess childhood-onset autoimmune diseases, it limits our ability to evaluate outcomes that typically manifest later in older adolescents or adulthood, such as autoimmune thyroid disorders. Thus, longer follow-up will be needed to assess the potential impact on these late-onset conditions. Sixth, this study was designed to assess the overall impact of antibiotic exposure during pregnancy and infancy on autoimmune disease risk. While subgroup analyses provided insights into potential differences among specific subgroups, these findings are hypothesis-generating rather than definitive. The effects of specific antibiotic categories on distinct autoimmune diseases warrant further investigation, and additional studies are needed to explore these associations in greater detail. Lastly, although the NHIS–NHID provides comprehensive data on the Korean population, differences in race and ethnicity, healthcare systems, and antibiotic use patterns may limit the generalizability of our findings to other settings.

In conclusion, our findings suggest no association between antibiotic exposure during the prenatal period or early infancy and the development of autoimmune diseases in children. This observation contrasts with several previous studies reporting increased risks and underscores the importance of carefully accounting for the underlying indications for antibiotic use and familial genetic susceptibility when interpreting such associations. While the potential benefits of antibiotic treatment in managing infections during pregnancy or early infancy likely outweigh the minimal risk of autoimmune outcomes, our findings also highlight the need for cautious and clinically appropriate antibiotic use during these critical developmental periods, particularly in specific subgroups.

## Supporting information

S1 Table Codes used to define exclusion criteria, exposures, outcomes, and covariates. (DOCX)

S2 Table Classification of antibiotics. (DOCX)

S3 Table Subgroup analyses of the risk of autoimmune disease associated with antibiotic exposure during pregnancy, according to antibiotic spectrum (broad-spectrum versus narrow-spectrum). (DOCX)

S4 Table Subgroup analyses of the risk of autoimmune disease associated with antibiotic exposure during pregnancy, according to antibiotic subclasses. (DOCX)

S5 Table Subgroup analyses of the risk of autoimmune disease associated with antibiotic exposure during pregnancy, according to antibiotic cumulative dose. (DOCX)

S6 Table Subgroup analyses of the risk of autoimmune disease associated with antibiotic exposure during pregnancy, according to sex. (DOCX)

S7 Table Subgroup analyses of the risk of autoimmune disease associated with antibiotic exposure during pregnancy, according to antibiotic exposure timing during pregnancy. (DOCX)

S8 Table Subgroup analyses of the risk of autoimmune disease associated with antibiotic exposure during early infancy, according to antibiotic spectrum (broad-spectrum versus narrow-spectrum). (DOCX)

S9 Table Subgroup analyses of the risk of autoimmune disease associated with antibiotic exposure during early infancy, according to antibiotic subclasses. (DOCX)

S10 Table Subgroup analyses of the risk of autoimmune disease associated with antibiotic exposure during early infancy, according to antibiotic cumulative dose. (DOCX)

S11 Table Subgroup analyses of the risk of autoimmune disease associated with antibiotic exposure during early infancy, according to sex. (DOCX)

S12 Table Subgroup analyses of the risk of autoimmune disease associated with antibiotic exposure during early infancy, according to antibiotic exposure timing during early infancy. (DOCX)

S13 Table Sensitivity analyses of the risk of autoimmune disease associated with antibiotic exposure during pregnancy. (DOCX)

S14 Table Sensitivity analyses of the risk of autoimmune disease associated with antibiotic exposure during early infancy. (DOCX)

S1 Protocol Summary Protocol. (DOCX)

S1 STROBE Checklist STROBE, Strengthening the Reporting of Observational Studies in Epidemiology. (DOCX)

## Abbreviations

IPTW: inverse probability of treatment weighting
NHIS–NHID: National Health Insurance Service–National Health Insurance Database
STROBE: Strengthening the Reporting of Observational Studies in Epidemiology
LMP: last menstrual period
BMI: body mass index
PS: propensity score
SMD: standardized mean difference
CI: confidence interval
HRs: Hazard ratios
NHIS: National Health Insurance Service
IBD: inflammatory bowel disease.
