# Supplementary material for: Exposure to antibiotics during pregnancy or early infancy and the risk of autoimmune disease in children: A nationwide cohort study in Korea
Source: PLoS Med. 2025 Aug 21;22(8):e1004677. doi: 10.1371/journal.pmed.1004677 (PMC12370083; doi:10.1371/journal.pmed.1004677)
Supplement: S1 Table — (DOCX) [file pmed.1004677.s001.docx]

**S1 Table.** Codes used to define exclusion criteria, exposures, outcomes, and covariates

| **Category** | **Definitions** |
| --- | --- |
| **Exclusion criteria** | **ICD-10 code** |
| Chromosomal abnormalities | Q90-Q99 |
| **Exposure** | **ATC code** |
| Antibiotics | J01 |
| **Outcome** | **ICD-10 code**  (defined as 2 outpatient visits or 1 inpatient visit) |
| Type 1 diabetes | E10 |
| Juvenile idiopathic arthritis | M08 |
| Ulcerative colitis | K51 |
| Crohn’s disease | K50 |
| Systemic lupus erythematosus | M32 |
| Hashimoto’s thyroiditis | E063 |
| **Cause of infection** | **ICD-10 code** |
| Respiratory infection | J00-J06, J13-J18, J20- J22, J32, J34.9, J35.0, J36, J39.0, J39.1, J40, J69, J85-J86 |
| Genitourinary infection | N10-N12, N13.6, N15.1, N15.9, N16.0, N30, N34, N37.0, N39.0, N70-N77, O23, O41.1, O98 |
| Gastrointestinal/Abdominal infection | A00–A07, A09, K35, K57, K61, K63.0, K65, K67, K75.0, K80.0, K80.1, K80.3, K80.4, K81.0, K81.9, K83.0 |
| Sexually Transmitted infection | A50-A64, A65–A69, A70–A74 |
| Skin, cutaneous, and mucosal infection | H00, H05.0, H44.0, H60.0–H60.3, H65-H66, J34.0, K12.2, L00-L08, L30.3, L66.3, L70, M72.6, N61, T81.4 |
| Other infections | Eye infection: H10, H13.1, H16, H19.1-H19.2  CNS infection: G00- G02, G04-G07  Musculoskeletal infection: M00- M01, M03, M46.2, M86  Orthopedic infections: M00, T845, M72.6, M60.0, M65.0–M65.1, M71.0–M71.1, M46.2–M46.5, M86  Miscellaneous bacterial infections: A15–A19, A20-A28, A30–A37, A38, A39–A49, A75–A79, H70, I33, K10.2, T82.6-T82.7, T85.7 |
| **Maternal comorbid conditions** | **ICD-10 code** |
| Asthma | J45-J46 |
| Chronic hypertension | I10-I15, O10 |
| Depression/mood disorder | F32-F39 |
| Endometriosis | N80.9 |
| Polycystic ovarian syndrome | E28.2 |
| Renal disease | E11.2, E13.2, E14.2, I12-I13, N00-N08, N17-N19, N25-N27 |
| Rheumatic disease | M05, M06, M07.0-M07.3, M08, L40.5, M32, M34.0, M34.1, M34.8, M34.9, M35.0, M35.2, M35.3, M45 |
| Migraine | G43 |
| GI disorder | K20, K21, K227, K25-K30, R12, E164, B980 |
| Anemia | D50, D51, D52, D53, D55, D56, D57, D58, D59.0, D59.1, D59.2, D59.4, D59.5, D59.6, D59.7, D59.8, D59.9, D60.0, D60.8, D60.9, D61, D63, D64 |
| **Maternal medication conditions** | **ATC code** |
| Acetaminophen | N02BE01 |
| Antiepileptic drugs | N03A (excl. N03AE) |
| Antidepressants | N06A |
| Antihypertensives | C03A, C03B, C03C, C03D, C03E, C07, C08C, C08D, C09A, C09B, C09C, C09D |
| Benzodiazepines | N03AE, N05BA, N05CD |
| NSAIDs | M01A |
| Fertility drugs | G03G |
| Thyroid medications | H03A |
| Lipid-lowering drugs | C10 |
| Antiacids | A02A |
| Systemic corticosteroids | H02AB |
| Triptans | N02CC |
| Antiemetics | A04A |
| COPD drugs | R03, R06 |
| Immunomodulator | L04AC, L04AD, L04AX |
| **Obstetric conditions** | **Procedure codes/ICD-10 codes** |
| Nulliparity | R3131, R3133, R3141, R3143, R4351, R4353, R4361, R4517, R4519, R4507, R4509, R5001, RA361, RA311, RA312, RA315, RA316, RA431, RA432 |
| Multiple gestations | R3133, R3138, R3143, R3148, R4353, R4358, R4516, R4519, R4520, R5001, R5002, RA312, RA314, RA316, RA318, RA432, RA434 |
| Preterm birth | O42, O60.1, O60.3 on mothers’ code at delivery date  P07.2, P07.3 on infants’ codes between delivery and delivery + 30 days |
| Cesarean section | R4514, R4516, R4517, R4518, R4519, R4520, R4507, R4508, R4509, R4510, R5001, R5002 |

**Abbreviations:** ATC, Anatomical Therapeutic Chemical Classification; ICD-10, International Classification of Diseases 10th revision.
