# Supplementary material for: Exposure to antibiotics during pregnancy or early infancy and the risk of autoimmune disease in children: A nationwide cohort study in Korea
Source: PLoS Med. 2025 Aug 21;22(8):e1004677. doi: 10.1371/journal.pmed.1004677 (PMC12370083; doi:10.1371/journal.pmed.1004677)
Supplement: S2 Table — (DOCX) [file pmed.1004677.s002.docx]

**S2 Table.** Classification of antibiotics

| **Antibiotic groups (ATC codes)** | **Specific medication** |
| --- | --- |
| Tetracyclines (J01AA) | doxycycline, oxytetracycline, tetracycline, minocycline, tigecycline |
| Penicillins (J01CA, J01CE and J01CF) | ampicillin, pivampicillin, amoxicillin, bacampicillin, pivmecillinam, azlocillin, mezlocillin, mecillinam, piperacillin, ticarcillin, metampicillin, talampicillin, sulbenicillin, sultamicillin, ciclacillin, benzylpenicillin, phenoxymethylpenicillin, benzathine, dicloxacillin, cloxacillin, methicillin, flucloxacillin, nafcillin |
| Cephalosporins (J01DB, J01DC, J01DD, and J01DE) | cephalexin, cefazolin, cefadroxil, cefazedone, cefatrizine, cefradine, cefroxadine, ceftezole, cefoxitin, cefuroxime, cefamandole, cefaclor, cefotetan, cefonicid, cefotiam, loracarbef, cefmetazole, cefprozil, ceforanide, cefminox, cefbuperazone, flomoxef, cefotaxime, ceftazidime, cefsulodin, ceftriaxone, cefmenoxime, ceftizoxime, cefixime, cefodizime, cefetamet, cefpiramide, cefoperazone, cefpodoxime, ceftibuten, ceftibuten, cefditoren, cefcapene, cefteram, cefepime, cefpirome |
| Sulfonamides and trimethoprim (J01EE) | sulfadiazine/trimethoprim, sulfamethoxazole/trimethoprim |
| Macrolides (J01FA) | erythromycin, spiramycin, midecamycin, roxithromycin, josamycin, clarithromycin, azithromycin, rokitamycin, dirithromycin, telithromycin, kitasamycin, acetylkitasamycin, acetylspiramycin |
| Lincosamides (J01FF) | clindamycin, lincomycin |
| Aminoglycosides (J01G) | streptomycin, tobramycin, gentamicin, kanamycin, neomycin, amikacin, netilmicin, sisomicin, dibekacin, ribostamycin, isepamicin, arbekacin, micronomicin, astromycin, astromicin, spectinomycin |
| Fluoroquinolones (J01MA) | zabofloxacin, balofloxacin, ofloxacin, ciprofloxacin, pefloxacin, enoxacin, norfloxacin, lomefloxacin, fleroxacin, sparfloxacin, rufloxacin, grepafloxacin, levofloxacin, moxifloxacin, gemifloxacin, gatifloxacin, tosufloxacin |
| Polymyxins (J01XB) | colistin, polymyxin B |
| Imidazole derivatives (J01XD) | metronidazole, tinidazole, ornidazole |
| Fosfomycin (J01XX01) | fosfomycin |
| Other antibiotic classes | Amphenicols (J01BA): chloramphenicol, thiamphenicol Monobactams (J01DF): aztreonam, carumonam Carbapenems (J01DH): meropenem, ertapenem, doripenem, imipenem, panipenem Streptogramins (J01FG): quinupristin Glycopeptides (J01XA): vancomycin, teicoplanin Steroid antibacterials (J01XC): fusidic acid Others: Nitrofurantoin (J01XE), Linezolid (J01XX08), Daptomycin (J01XX09), Bacitracin (J01XX10), Tedizolid (J01XX11) |

**Abbreviations:** ATC, Anatomical Therapeutic Chemical Classification.
