# Supplementary material for: Exposure to antibiotics during pregnancy or early infancy and the risk of autoimmune disease in children: A nationwide cohort study in Korea
Source: PLoS Med. 2025 Aug 21;22(8):e1004677. doi: 10.1371/journal.pmed.1004677 (PMC12370083; doi:10.1371/journal.pmed.1004677)
Supplement: S3 Table — (DOCX) [file pmed.1004677.s003.docx]

**S3 Table.** Subgroup analyses of risk of autoimmune disease associated with antibiotic exposure during pregnancy according to antibiotic **antibiotic spectrum (broad-spectrum vs. narrow-spectrum)**

| **Spectrum** | **Outcome** | **Exposure** | **No_Patients** | **No_Events** | **IRper100000PY** | **aHR** | **95% CI** |
| --- | --- | --- | --- | --- | --- | --- | --- |
| Broad | T1D | Exposed | 1130490 | 328 | 4.03 | 1.17 | 0.98 to 1.40 |
|  |  | Unexposed | 1025724 | 273 | 3.57 |  |  |
|  | JIA | Exposed | 1130490 | 274 | 3.37 | 1.02 | 0.84 to 1.23 |
|  |  | Unexposed | 1025724 | 260 | 3.40 |  |  |
|  | UC | Exposed | 1130490 | 116 | 1.42 | 1.03 | 0.75 to 1.40 |
|  |  | Unexposed | 1025724 | 95 | 1.24 |  |  |
|  | CD | Exposed | 1130490 | 389 | 4.78 | 1.19 | 1.00 to 1.42 |
|  |  | Unexposed | 1025724 | 303 | 3.97 |  |  |
|  | SLE | Exposed | 1130490 | 66 | 0.81 | 0.73 | 0.50 to 1.06 |
|  |  | Unexposed | 1025724 | 70 | 0.92 |  |  |
|  | HT | Exposed | 1130490 | 413 | 5.07 | 1.15 | 0.98 to 1.35 |
|  |  | Unexposed | 1025724 | 336 | 4.40 |  |  |
| Narrow | T1D | Exposed | 293830 | 91 | 3.95 | 1.04 | 0.77 to 1.39 |
|  |  | Unexposed | 1024465 | 290 | 3.72 |  |  |
|  | JIA | Exposed | 293830 | 93 | 4.04 | 1.33 | 0.99 to 1.81 |
|  |  | Unexposed | 1024465 | 269 | 3.45 |  |  |
|  | UC | Exposed | 293830 | 41 | 1.78 | 1.43 | 0.90 to 2.27 |
|  |  | Unexposed | 1024465 | 100 | 1.28 |  |  |
|  | CD | Exposed | 293830 | 106 | 4.60 | 1.04 | 0.79 to 1.37 |
|  |  | Unexposed | 1024465 | 318 | 4.07 |  |  |
|  | SLE | Exposed | 293830 | 14 | 0.61 | 0.56 | 0.26 to 1.20 |
|  |  | Unexposed | 1024465 | 75 | 0.96 |  |  |
|  | HT | Exposed | 293830 | 103 | 4.47 | 0.89 | 0.67 to 1.18 |
|  |  | Unexposed | 1024465 | 351 | 4.50 |  |  |

**Abbreviation:** aHR, adjusted hazard ratio; CD, Crohn's disease; CI, confidence interval; IR, incidence rate; HT, Hashimoto’s thyroiditis; JIA, juvenile idiopathic arthritis; T1D, type 1 diabetes; PY, person-year; UC, ulcerative colitis; SLE, systemic lupus erythematosus.
