# Supplementary material for: Exposure to antibiotics during pregnancy or early infancy and the risk of autoimmune disease in children: A nationwide cohort study in Korea
Source: PLoS Med. 2025 Aug 21;22(8):e1004677. doi: 10.1371/journal.pmed.1004677 (PMC12370083; doi:10.1371/journal.pmed.1004677)
Supplement: S4 Table — (DOCX) [file pmed.1004677.s004.docx]

**S4 Table.** Subgroup analyses of risk of autoimmune disease associated with antibiotic exposure during pregnancy according to antibiotic **subclasses**

| **Subclasses** | **Outcome** | **exposure** | **No_Patients** | **No_Events** | **IRper100000PY** | **aHR** | **95% CI** |
| --- | --- | --- | --- | --- | --- | --- | --- |
| Cephalosporins | T1D | Exposed | 735814 | 199 | 3.71 | 0.99 | 0.81 to 1.23 |
|  |  | Unexposed | 1026394 | 277 | 3.61 |  |  |
|  | JIA | Exposed | 735814 | 183 | 3.41 | 0.98 | 0.79 to 1.21 |
|  |  | Unexposed | 1026394 | 263 | 3.42 |  |  |
|  | UC | Exposed | 735814 | 79 | 1.47 | 1.01 | 0.72 to 1.41 |
|  |  | Unexposed | 1026394 | 98 | 1.28 |  |  |
|  | CD | Exposed | 735814 | 267 | 4.97 | 1.29 | 1.07 to 1.57 |
|  |  | Unexposed | 1026394 | 310 | 4.04 |  |  |
|  | SLE | Exposed | 735814 | 44 | 0.82 | 0.72 | 0.47 to 1.10 |
|  |  | Unexposed | 1026394 | 71 | 0.92 |  |  |
|  | HT | Exposed | 735814 | 270 | 5.03 | 1.05 | 0.88 to 1.27 |
|  |  | Unexposed | 1026394 | 345 | 4.49 |  |  |
| Imidazoles | T1D | Exposed | 115795 | 24 | 3.25 | 1.36 | 0.76 to 2.44 |
|  |  | Unexposed | 942760 | 243 | 3.55 |  |  |
|  | JIA | Exposed | 115795 | 25 | 3.38 | 0.99 | 0.60 to 1.65 |
|  |  | Unexposed | 942760 | 228 | 3.33 |  |  |
|  | UC | Exposed | 115795 | 11 | 1.49 | 1.10 | 0.48 to 2.52 |
|  |  | Unexposed | 942760 | 91 | 1.33 |  |  |
|  | CD | Exposed | 115795 | 34 | 4.60 | 1.07 | 0.65 to 1.77 |
|  |  | Unexposed | 942760 | 270 | 3.94 |  |  |
|  | SLE | Exposed | 115795 | 3 | 0.41 | 0.19 | 0.05 to 0.65 |
|  |  | Unexposed | 942760 | 67 | 0.98 |  |  |
|  | HT | Exposed | 115795 | 26 | 3.52 | 1.01 | 0.57 to 1.78 |
|  |  | Unexposed | 942760 | 296 | 4.32 |  |  |
| Macrolides | T1D | Exposed | 210926 | 50 | 3.74 | 1.14 | 0.6 to 1.71 |
|  |  | Unexposed | 988505 | 254 | 3.55 |  |  |
|  | JIA | Exposed | 210926 | 40 | 2.99 | 0.77 | 0.49 to 1.21 |
|  |  | Unexposed | 988505 | 239 | 3.34 |  |  |
|  | UC | Exposed | 210926 | 12 | 0.90 | 0.69 | 0.30 to 1.60 |
|  |  | Unexposed | 988505 | 95 | 1.33 |  |  |
|  | CD | Exposed | 210926 | 52 | 3.89 | 1.17 | 0.79 to 1.72 |
|  |  | Unexposed | 988505 | 278 | 3.88 |  |  |
|  | SLE | Exposed | 210926 | 15 | 1.12 | 1.00 | 0.49 to 2.05 |
|  |  | Unexposed | 988505 | 69 | 0.96 |  |  |
|  | HT | Exposed | 210926 | 57 | 4.26 | 1.00 | 0.69 to 1.45 |
|  |  | Unexposed | 988505 | 310 | 4.33 |  |  |
| Penicillin | T1D | Exposed | 568417 | 177 | 4.16 | 1.19 | 0.95 to 1.50 |
|  |  | Unexposed | 1003631 | 277 | 3.66 |  |  |
|  | JIA | Exposed | 568417 | 147 | 3.45 | 1.17 | 0.91 to 1.51 |
|  |  | Unexposed | 1003631 | 261 | 3.45 |  |  |
|  | UC | Exposed | 568417 | 69 | 1.62 | 1.24 | 0.85 to 1.80 |
|  |  | Unexposed | 1003631 | 96 | 1.27 |  |  |
|  | CD | Exposed | 568417 | 187 | 4.39 | 1.07 | 0.85 to 1.33 |
|  |  | Unexposed | 1003631 | 306 | 4.04 |  |  |
|  | SLE | Exposed | 568417 | 33 | 0.77 | 0.65 | 0.39 to 1.08 |
|  |  | Unexposed | 1003631 | 71 | 0.94 |  |  |
|  | HT | Exposed | 568417 | 195 | 4.58 | 1.02 | 0.82 to 1.27 |
|  |  | Unexposed | 1003631 | 339 | 4.48 |  |  |

**Abbreviation:** aHR, adjusted hazard ratio; CD, Crohn's disease; CI, confidence interval; IR, incidence rate; HT, Hashimoto’s thyroiditis; JIA, juvenile idiopathic arthritis; T1D, type 1 diabetes; PY, person-year; UC, ulcerative colitis; SLE, systemic lupus erythematosus.
