# Supplementary material for: Exposure to antibiotics during pregnancy or early infancy and the risk of autoimmune disease in children: A nationwide cohort study in Korea
Source: PLoS Med. 2025 Aug 21;22(8):e1004677. doi: 10.1371/journal.pmed.1004677 (PMC12370083; doi:10.1371/journal.pmed.1004677)
Supplement: S5 Table — (DOCX) [file pmed.1004677.s005.docx]

**S5 Table.** Subgroup analyses of risk of autoimmune disease associated with antibiotic exposure during pregnancy according to antibiotic **cumulative dose**

| **Cumulative dose** | **Outcome** | **Exposure** | **No_Patients** | **No_Events** | **IRper100000PY** | **aHR** | **95% CI** |
| --- | --- | --- | --- | --- | --- | --- | --- |
| <4 days | T1D | Exposed | 577159 | 170 | 3.98 | 1.13 | 0.92 to 1.38 |
|  |  | Unexposed | 1067048 | 293 | 3.68 |  |  |
|  | JIA | Exposed | 577159 | 135 | 3.16 | 0.97 | 0.78 to 1.21 |
|  |  | Unexposed | 1067048 | 271 | 3.40 |  |  |
|  | UC | Exposed | 577159 | 60 | 1.41 | 1.10 | 0.78 to 1.54 |
|  |  | Unexposed | 1067048 | 99 | 1.24 |  |  |
|  | CD | Exposed | 577159 | 190 | 4.45 | 1.11 | 0.91 to 1.35 |
|  |  | Unexposed | 1067048 | 322 | 4.04 |  |  |
|  | SLE | Exposed | 577159 | 32 | 0.75 | 0.74 | 0.47 to 1.15 |
|  |  | Unexposed | 1067048 | 76 | 0.95 |  |  |
|  | HT | Exposed | 577159 | 197 | 4.61 | 1.04 | 0.86 to 1.25 |
|  |  | Unexposed | 1067048 | 351 | 4.40 |  |  |
| 4-7 days | T1D | Exposed | 361778 | 106 | 4.01 | 1.06 | 0.82 to 1.38 |
|  |  | Unexposed | 1048951 | 280 | 3.57 |  |  |
|  | JIA | Exposed | 361778 | 88 | 3.33 | 1.06 | 0.80 to 1.42 |
|  |  | Unexposed | 1048951 | 267 | 3.41 |  |  |
|  | UC | Exposed | 361778 | 37 | 1.40 | 0.99 | 0.64 to 1.52 |
|  |  | Unexposed | 1048951 | 99 | 1.26 |  |  |
|  | CD | Exposed | 361778 | 152 | 5.75 | 1.30 | 1.04 to 1.63 |
|  |  | Unexposed | 1048951 | 315 | 4.02 |  |  |
|  | SLE | Exposed | 361778 | 21 | 0.79 | 0.71 | 0.41 to 1.23 |
|  |  | Unexposed | 1048951 | 73 | 0.93 |  |  |
|  | HT | Exposed | 361778 | 132 | 4.99 | 1.08 | 0.86 to 1.37 |
|  |  | Unexposed | 1048951 | 350 | 4.47 |  |  |
| 8-12 days | T1D | Exposed | 241386 | 69 | 3.96 | 1.22 | 0.82 to 1.82 |
|  |  | Unexposed | 914807 | 239 | 3.51 |  |  |
|  | JIA | Exposed | 241386 | 64 | 3.68 | 1.13 | 0.80 to 1.60 |
|  |  | Unexposed | 914807 | 224 | 3.29 |  |  |
|  | UC | Exposed | 241386 | 26 | 1.49 | 1.09 | 0.63 to 1.89 |
|  |  | Unexposed | 914807 | 89 | 1.31 |  |  |
|  | CD | Exposed | 241386 | 68 | 3.91 | 1.18 | 0.80 to 1.74 |
|  |  | Unexposed | 914807 | 273 | 4.01 |  |  |
|  | SLE | Exposed | 241386 | 15 | 0.86 | 0.55 | 0.28 to 1.06 |
|  |  | Unexposed | 914807 | 67 | 0.98 |  |  |
|  | HT | Exposed | 241386 | 94 | 5.40 | 1.22 | 0.87 to 1.70 |
|  |  | Unexposed | 914807 | 302 | 4.44 |  |  |
| +13 days | T1D | Exposed | 149907 | 39 | 3.66 | 0.90 | 0.50 to 1.63 |
|  |  | Unexposed | 823421 | 218 | 3.58 |  |  |
|  | JIA | Exposed | 149907 | 31 | 2.91 | 0.94 | 0.52 to 1.71 |
|  |  | Unexposed | 823421 | 210 | 3.44 |  |  |
|  | UC | Exposed | 149907 | 15 | 1.41 | 0.93 | 0.41 to 2.10 |
|  |  | Unexposed | 823421 | 80 | 1.31 |  |  |
|  | CD | Exposed | 149907 | 53 | 4.98 | 1.50 | 0.94 to 2.38 |
|  |  | Unexposed | 823421 | 243 | 3.99 |  |  |
|  | SLE | Exposed | 149907 | 6 | 0.56 | 0.18 | 0.07 to 0.44 |
|  |  | Unexposed | 823421 | 62 | 1.02 |  |  |
|  | HT | Exposed | 149907 | 54 | 5.07 | 1.01 | 0.60 to 1.68 |
|  |  | Unexposed | 823421 | 271 | 4.45 |  |  |

**Abbreviation:** aHR, adjusted hazard ratio; CD, Crohn's disease; CI, confidence interval; IR, incidence rate; HT, Hashimoto’s thyroiditis; JIA, juvenile idiopathic arthritis; T1D, type 1 diabetes; PY, person-year; UC, ulcerative colitis; SLE, systemic lupus erythematosus.
