# Supplementary material for: Exposure to antibiotics during pregnancy or early infancy and the risk of autoimmune disease in children: A nationwide cohort study in Korea
Source: PLoS Med. 2025 Aug 21;22(8):e1004677. doi: 10.1371/journal.pmed.1004677 (PMC12370083; doi:10.1371/journal.pmed.1004677)
Supplement: S6 Table — (DOCX) [file pmed.1004677.s006.docx]

**S6 Table.** Subgroup analyses of risk of autoimmune disease associated with antibiotic exposure during pregnancy according to **sex**

| **Sex** | **Outcome** | **Exposure** | **No_Patients** | **No_Events** | **IRper100000PY** | **aHR** | **95% CI** |
| --- | --- | --- | --- | --- | --- | --- | --- |
| Male | T1D | Exposed | 688767 | 171 | 3.40 | 1.15 | 0.88 to 1.50 |
|  |  | Unexposed | 532768 | 117 | 2.94 |  |  |
|  | JIA | Exposed | 688767 | 152 | 3.02 | 1.09 | 0.83 to 1.43 |
|  |  | Unexposed | 532768 | 113 | 2.84 |  |  |
|  | UC | Exposed | 688767 | 72 | 1.43 | 0.82 | 0.55 to 1.23 |
|  |  | Unexposed | 532768 | 58 | 1.46 |  |  |
|  | CD | Exposed | 688767 | 272 | 5.40 | 1.17 | 0.94 to 1.44 |
|  |  | Unexposed | 532768 | 185 | 4.64 |  |  |
|  | SLE | Exposed | 688767 | 28 | 0.56 | 0.61 | 0.34 to 1.09 |
|  |  | Unexposed | 532768 | 29 | 0.73 |  |  |
|  | HT | Exposed | 688767 | 100 | 1.99 | 0.98 | 0.69 to 1.39 |
|  |  | Unexposed | 532768 | 68 | 1.71 |  |  |
| Female | T1D | Exposed | 509100 | 217 | 4.58 | 1.14 | 0.91 to 1.42 |
|  |  | Unexposed | 648852 | 162 | 4.26 |  |  |
|  | JIA | Exposed | 509100 | 174 | 3.67 | 0.95 | 0.74 to 1.21 |
|  |  | Unexposed | 648852 | 152 | 3.00 |  |  |
|  | UC | Exposed | 509100 | 65 | 1.37 | 1.36 | 0.89 to 2.07 |
|  |  | Unexposed | 648852 | 42 | 1.10 |  |  |
|  | CD | Exposed | 509100 | 196 | 4.14 | 1.16 | 0.91 to 1.50 |
|  |  | Unexposed | 648852 | 128 | 3.37 |  |  |
|  | SLE | Exposed | 509100 | 48 | 1.01 | 0.76 | 0.48 to 1.18 |
|  |  | Unexposed | 648852 | 43 | 1.13 |  |  |
|  | HT | Exposed | 509100 | 379 | 8.00 | 1.09 | 0.92 to 1.29 |
|  |  | Unexposed | 648852 | 278 | 7.31 |  |  |

**Abbreviation:** aHR, adjusted hazard ratio; CD, Crohn's disease; CI, confidence interval; IR, incidence rate; HT, Hashimoto’s thyroiditis; JIA, juvenile idiopathic arthritis; T1D, type 1 diabetes; PY, person-year; UC, ulcerative colitis; SLE, systemic lupus erythematosus.
