# Supplementary material for: Exposure to antibiotics during pregnancy or early infancy and the risk of autoimmune disease in children: A nationwide cohort study in Korea
Source: PLoS Med. 2025 Aug 21;22(8):e1004677. doi: 10.1371/journal.pmed.1004677 (PMC12370083; doi:10.1371/journal.pmed.1004677)
Supplement: S7 Table — (DOCX) [file pmed.1004677.s007.docx]

**S7 Table.** Subgroup analyses of risk of autoimmune disease associated with antibiotic exposure during pregnancy according to antibiotic **exposure timing** during pregnancy

| **Timing** | **Outcome** | **exposure** | **No_Patients** | **No_Events** | **IRper100000PY** | **aHR** | **95% CI** |
| --- | --- | --- | --- | --- | --- | --- | --- |
| 1st trimester | T1D | Exposed | 728142 | 211 | 3.98 | 1.06 | 0.86 to 1.30 |
|  |  | Unexposed | 991145 | 263 | 3.56 |  |  |
|  | JIA | Exposed | 728142 | 169 | 3.19 | 0.99 | 0.79 to 1.24 |
|  |  | Unexposed | 991145 | 256 | 3.46 |  |  |
|  | UC | Exposed | 728142 | 85 | 1.60 | 1.16 | 0.82 to 1.64 |
|  |  | Unexposed | 991145 | 93 | 1.26 |  |  |
|  | CD | Exposed | 728142 | 264 | 4.98 | **1.27** | **1.04 to 1.54** |
|  |  | Unexposed | 991145 | 294 | 3.97 |  |  |
|  | SLE | Exposed | 728142 | 38 | 0.72 | 0.61 | 0.39 to 0.94 |
|  |  | Unexposed | 991145 | 67 | 0.91 |  |  |
|  | HT | Exposed | 728142 | 254 | 4.79 | 1.00 | 0.83 to 1.22 |
|  |  | Unexposed | 991145 | 328 | 4.43 |  |  |
| 2nd trimester | T1D | Exposed | 559800 | 148 | 3.63 | 1.14 | 0.90 to 1.44 |
|  |  | Unexposed | 1015751 | 271 | 3.56 |  |  |
|  | JIA | Exposed | 559800 | 135 | 3.31 | 1.04 | 0.81 to 1.32 |
|  |  | Unexposed | 1015751 | 260 | 3.42 |  |  |
|  | UC | Exposed | 559800 | 65 | 1.59 | 1.04 | 0.72 to 1.49 |
|  |  | Unexposed | 1015751 | 98 | 1.29 |  |  |
|  | CD | Exposed | 559800 | 213 | 5.22 | **1.27** | **1.03 to 1.57** |
|  |  | Unexposed | 1015751 | 303 | 3.98 |  |  |
|  | SLE | Exposed | 559800 | 34 | 0.83 | 0.67 | 0.42 to 1.04 |
|  |  | Unexposed | 1015751 | 70 | 0.92 |  |  |
|  | HT | Exposed | 559800 | 212 | 5.20 | 1.15 | 0.94 to 1.40 |
|  |  | Unexposed | 1015751 | 342 | 4.50 |  |  |
| 3rd trimester | T1D | Exposed | 629859 | 173 | 3.76 | 1.09 | 0.87 to 1.35 |
|  |  | Unexposed | 1035395 | 276 | 3.57 |  |  |
|  | JIA | Exposed | 629859 | 167 | 3.63 | 1.07 | 0.85 to 1.33 |
|  |  | Unexposed | 1035395 | 262 | 3.39 |  |  |
|  | UC | Exposed | 629859 | 57 | 1.24 | 0.94 | 0.65 to 1.35 |
|  |  | Unexposed | 1035395 | 100 | 1.29 |  |  |
|  | CD | Exposed | 629859 | 203 | 4.41 | 1.16 | 0.95 to 1.42 |
|  |  | Unexposed | 1035395 | 311 | 4.03 |  |  |
|  | SLE | Exposed | 629859 | 36 | 0.78 | 0.78 | 0.49 to 1.25 |
|  |  | Unexposed | 1035395 | 73 | 0.95 |  |  |
|  | HT | Exposed | 629859 | 239 | 5.19 | 1.16 | 0.96 to 1.41 |
|  |  | Unexposed | 1035395 | 344 | 4.45 |  |  |

**Abbreviation:** aHR, adjusted hazard ratio; CD, Crohn's disease; CI, confidence interval; IR, incidence rate; HT, Hashimoto’s thyroiditis; JIA, juvenile idiopathic arthritis; T1D, type 1 diabetes; PY, person-year; UC, ulcerative colitis; SLE, systemic lupus erythematosus.
