# Supplementary material for: Exposure to antibiotics during pregnancy or early infancy and the risk of autoimmune disease in children: A nationwide cohort study in Korea
Source: PLoS Med. 2025 Aug 21;22(8):e1004677. doi: 10.1371/journal.pmed.1004677 (PMC12370083; doi:10.1371/journal.pmed.1004677)
Supplement: S8 Table — (DOCX) [file pmed.1004677.s008.docx]

**S8 Table.** Subgroup analyses of risk of autoimmune disease associated with antibiotic exposure during early infancy according to antibiotic **antibiotic spectrum (broad-spectrum vs. narrow-spectrum)**

| **Spectrum** | **Outcome** | **exposure** | **No_Patients** | **No_Events** | **IRper100000PY** | **aHR** | **95% CI** |
| --- | --- | --- | --- | --- | --- | --- | --- |
| Broad | T1D | Exposed | 1336969 | 385 | 3.42 | 1.03 | 0.86 to 1.24 |
|  |  | Unexposed | 1283533 | 336 | 3.29 |  |  |
|  | JIA | Exposed | 1336969 | 356 | 3.16 | 1.08 | 0.89 to 1.30 |
|  |  | Unexposed | 1283533 | 293 | 2.87 |  |  |
|  | UC | Exposed | 1336969 | 93 | 0.83 | 0.94 | 0.65 to 1.35 |
|  |  | Unexposed | 1283533 | 84 | 0.82 |  |  |
|  | CD | Exposed | 1336969 | 491 | 4.36 | 1.09 | 0.93 to 1.28 |
|  |  | Unexposed | 1283533 | 385 | 3.77 |  |  |
|  | SLE | Exposed | 1336969 | 79 | 0.70 | 1.32 | 0.83 to 2.10 |
|  |  | Unexposed | 1283533 | 49 | 0.48 |  |  |
|  | HT | Exposed | 1336969 | 489 | 4.34 | 1.09 | 0.93 to 1.28 |
|  |  | Unexposed | 1283533 | 406 | 3.97 |  |  |
| Narrow | T1D | Exposed | 474174 | 136 | 3.41 | 1.11 | 0.85 to 1.45 |
|  |  | Unexposed | 1282793 | 334 | 3.28 |  |  |
|  | JIA | Exposed | 474174 | 142 | 3.56 | 1.21 | 0.93 to 1.59 |
|  |  | Unexposed | 1282793 | 288 | 2.83 |  |  |
|  | UC | Exposed | 474174 | 36 | 0.90 | 1.04 | 0.60 to 1.78 |
|  |  | Unexposed | 1282793 | 84 | 0.83 |  |  |
|  | CD | Exposed | 474174 | 179 | 4.48 | 1.05 | 0.82 to 1.32 |
|  |  | Unexposed | 1282793 | 390 | 3.83 |  |  |
|  | SLE | Exposed | 474174 | 30 | 0.75 | 1.21 | 0.81 to 1.83 |
|  |  | Unexposed | 1282793 | 48 | 0.47 |  |  |
|  | HT | Exposed | 474174 | 213 | 5.34 | 1.13 | 0.97 to 1.31 |
|  |  | Unexposed | 1282793 | 403 | 3.96 |  |  |

**Abbreviation:** aHR, adjusted hazard ratio; CD, Crohn's disease; CI, confidence interval; IR, incidence rate; HT, Hashimoto’s thyroiditis; JIA, juvenile idiopathic arthritis; T1D, type 1 diabetes; PY, person-year; UC, ulcerative colitis; SLE, systemic lupus erythematosus.
