# Supplementary material for: Exposure to antibiotics during pregnancy or early infancy and the risk of autoimmune disease in children: A nationwide cohort study in Korea
Source: PLoS Med. 2025 Aug 21;22(8):e1004677. doi: 10.1371/journal.pmed.1004677 (PMC12370083; doi:10.1371/journal.pmed.1004677)
Supplement: S9 Table — (DOCX) [file pmed.1004677.s009.docx]

**S9 Table.** Subgroup analyses of risk of autoimmune disease associated with antibiotic exposure during early infancy according to antibiotic **subclasses**

| **Subclasses** | **Outcome** | **exposure** | **No_Patients** | **No_Events** | **IRper100000PY** | **aHR** | **95% CI** |
| --- | --- | --- | --- | --- | --- | --- | --- |
| Cephalosporins | T1D | Exposed | 702070 | 229 | 3.87 | 1.25 | 0.99 to 1.55 |
|  |  | Unexposed | 1235812 | 327 | 3.31 |  |  |
|  | JIA | Exposed | 702070 | 181 | 3.06 | 1.05 | 0.84 to 1.33 |
|  |  | Unexposed | 1235812 | 283 | 2.86 |  |  |
|  | UC | Exposed | 702070 | 52 | 0.88 | 1.06 | 0.69 to 1.63 |
|  |  | Unexposed | 1235812 | 78 | 0.79 |  |  |
|  | CD | Exposed | 702070 | 263 | 4.45 | 1.07 | 0.88 to 1.31 |
|  |  | Unexposed | 1235812 | 375 | 3.80 |  |  |
|  | SLE | Exposed | 702070 | 42 | 0.71 | 1.23 | 0.99 to 1.51 |
|  |  | Unexposed | 1235812 | 47 | 0.48 |  |  |
|  | HT | Exposed | 702070 | 252 | 4.26 | 1.07 | 0.49 to 2.31 |
|  |  | Unexposed | 1235812 | 385 | 3.90 |  |  |
| Macrolides | T1D | Exposed | 446995 | 137 | 3.47 | 1.15 | 0.88 to 1.51 |
|  |  | Unexposed | 1119418 | 311 | 3.39 |  |  |
|  | JIA | Exposed | 446995 | 110 | 2.79 | 0.89 | 0.67 to 1.19 |
|  |  | Unexposed | 1119418 | 264 | 2.88 |  |  |
|  | UC | Exposed | 446995 | 37 | 0.94 | 1.34 | 0.75 to 2.40 |
|  |  | Unexposed | 1119418 | 73 | 0.80 |  |  |
|  | CD | Exposed | 446995 | 182 | 4.62 | 0.98 | 0.77 to 1.25 |
|  |  | Unexposed | 1119418 | 342 | 3.73 |  |  |
|  | SLE | Exposed | 446995 | 27 | 0.68 | 1.31 | 0.67 to 2.58 |
|  |  | Unexposed | 1119418 | 44 | 0.48 |  |  |
|  | HT | Exposed | 446995 | 189 | 4.79 | 0.98 | 0.85 to 1.14 |
|  |  | Unexposed | 1119418 | 358 | 3.90 |  |  |
| Penicillin | T1D | Exposed | 912089 | 244 | 3.22 | 1.01 | 0.82 to 1.23 |
|  |  | Unexposed | 1270291 | 331 | 3.28 |  |  |
|  | JIA | Exposed | 912089 | 248 | 3.27 | 1.17 | 0.95 to 1.44 |
|  |  | Unexposed | 1270291 | 286 | 2.83 |  |  |
|  | UC | Exposed | 912089 | 62 | 0.82 | 0.92 | 0.60 to 1.40 |
|  |  | Unexposed | 1270291 | 82 | 0.81 |  |  |
|  | CD | Exposed | 912089 | 320 | 4.22 | 1.05 | 0.88 to 1.26 |
|  |  | Unexposed | 1270291 | 382 | 3.79 |  |  |
|  | SLE | Exposed | 912089 | 44 | 0.58 | 1.27 | 0.74 to 2.17 |
|  |  | Unexposed | 1270291 | 47 | 0.47 |  |  |
|  | HT | Exposed | 912089 | 333 | 4.39 | 1.01 | 0.88 to 1.15 |
|  |  | Unexposed | 1270291 | 398 | 3.94 |  |  |

**Abbreviation:** aHR, adjusted hazard ratio; CD, Crohn's disease; CI, confidence interval; IR, incidence rate; HT, Hashimoto’s thyroiditis; JIA, juvenile idiopathic arthritis; T1D, type 1 diabetes; PY, person-year; UC, ulcerative colitis; SLE, systemic lupus erythematosus.
