# Supplementary material for: Exposure to antibiotics during pregnancy or early infancy and the risk of autoimmune disease in children: A nationwide cohort study in Korea
Source: PLoS Med. 2025 Aug 21;22(8):e1004677. doi: 10.1371/journal.pmed.1004677 (PMC12370083; doi:10.1371/journal.pmed.1004677)
Supplement: S10 Table — (DOCX) [file pmed.1004677.s010.docx]

**S10 Table.** Subgroup analyses of risk of autoimmune disease associated with antibiotic exposure during early infancy according to antibiotic **cumulative dose**

| **Cumulative dose** | **Outcome** | **exposure** | **No_Patients** | **No_Events** | **IRper100000PY** | **aHR** | **95% CI** |
| --- | --- | --- | --- | --- | --- | --- | --- |
| <8 days | T1D | Exposed | 766228 | 224 | 3.46 | 0.97 | 0.80 to 1.17 |
|  |  | Unexposed | 1330173 | 342 | 3.27 |  |  |
|  | JIA | Exposed | 766228 | 199 | 3.07 | 1.07 | 0.87 to 1.30 |
|  |  | Unexposed | 1330173 | 298 | 2.85 |  |  |
|  | UC | Exposed | 766228 | 48 | 0.74 | 0.93 | 0.63 to 1.37 |
|  |  | Unexposed | 1330173 | 86 | 0.82 |  |  |
|  | CD | Exposed | 766228 | 256 | 3.95 | 0.99 | 0.83 to 1.17 |
|  |  | Unexposed | 1330173 | 397 | 3.79 |  |  |
|  | SLE | Exposed | 766228 | 44 | 0.68 | 1.33 | 0.85 to 2.07 |
|  |  | Unexposed | 1330173 | 51 | 0.49 |  |  |
|  | HT | Exposed | 766228 | 276 | 4.26 | 1.06 | 0.89 to 1.25 |
|  |  | Unexposed | 1330173 | 414 | 3.96 |  |  |
| 8-16 days | T1D | Exposed | 388396 | 122 | 3.74 | 1.02 | 0.85 to 1.23 |
|  |  | Unexposed | 1188286 | 310 | 3.25 |  |  |
|  | JIA | Exposed | 388396 | 102 | 3.12 | 1.19 | 0.98 to 1.44 |
|  |  | Unexposed | 1188286 | 276 | 2.89 |  |  |
|  | UC | Exposed | 388396 | 28 | 0.86 | 0.95 | 0.65 to 1.40 |
|  |  | Unexposed | 1188286 | 76 | 0.80 |  |  |
|  | CD | Exposed | 388396 | 141 | 4.32 | 1.02 | 0.86 to 1.22 |
|  |  | Unexposed | 1188286 | 362 | 3.79 |  |  |
|  | SLE | Exposed | 388396 | 21 | 0.64 | 1.07 | 0.66 to 1.72 |
|  |  | Unexposed | 1188286 | 46 | 0.48 |  |  |
|  | HT | Exposed | 388396 | 127 | 3.89 | 0.92 | 0.77 to 1.09 |
|  |  | Unexposed | 1188286 | 371 | 3.89 |  |  |
| 17-28 days | T1D | Exposed | 164439 | 52 | 3.73 | 1.09 | 0.87 to 1.36 |
|  |  | Unexposed | 872494 | 242 | 3.41 |  |  |
|  | JIA | Exposed | 164439 | 51 | 3.66 | 1.14 | 0.90 to 1.44 |
|  |  | Unexposed | 872494 | 203 | 2.86 |  |  |
|  | UC | Exposed | 164439 | 14 | 1.00 | 1.09 | 0.69 to 1.70 |
|  |  | Unexposed | 872494 | 60 | 0.85 |  |  |
|  | CD | Exposed | 164439 | 78 | 5.59 | 0.93 | 1.07 to 1.56 |
|  |  | Unexposed | 872494 | 275 | 3.88 |  |  |
|  | SLE | Exposed | 164439 | 9 | 0.64 | 1.04 | 0.60 to 1.82 |
|  |  | Unexposed | 872494 | 37 | 0.52 |  |  |
|  | HT | Exposed | 164439 | 72 | 5.16 | 0.93 | 0.76 to 1.15 |
|  |  | Unexposed | 872494 | 283 | 3.99 |  |  |
| +29 days | T1D | Exposed | 64761 | 17 | 3.09 | 1.07 | 0.81 to 1.41 |
|  |  | Unexposed | 720410 | 194 | 3.35 |  |  |
|  | JIA | Exposed | 64761 | 18 | 3.27 | 1.24 | 0.93 to 1.64 |
|  |  | Unexposed | 720410 | 171 | 2.95 |  |  |
|  | UC | Exposed | 64761 | 4 | 0.73 | 1.04 | 0.59 to 1.83 |
|  |  | Unexposed | 720410 | 53 | 0.92 |  |  |
|  | CD | Exposed | 64761 | 22 | 4.00 | 1.24 | 0.93 to 1.64 |
|  |  | Unexposed | 720410 | 225 | 3.89 |  |  |
|  | SLE | Exposed | 64761 | 3 | 0.55 | 1.01 | 0.50 to 2.06 |
|  |  | Unexposed | 720410 | 22 | 0.38 |  |  |
|  | HT | Exposed | 64761 | 29 | 5.27 | 1.12 | 0.88 to 1.43 |
|  |  | Unexposed | 720410 | 205 | 3.54 |  |  |

**Abbreviation:** aHR, adjusted hazard ratio; CD, Crohn's disease; CI, confidence interval; IR, incidence rate; HT, Hashimoto’s thyroiditis; JIA, juvenile idiopathic arthritis; T1D, type 1 diabetes; PY, person-year; UC, ulcerative colitis; SLE, systemic lupus erythematosus.
