# Supplementary material for: Exposure to antibiotics during pregnancy or early infancy and the risk of autoimmune disease in children: A nationwide cohort study in Korea
Source: PLoS Med. 2025 Aug 21;22(8):e1004677. doi: 10.1371/journal.pmed.1004677 (PMC12370083; doi:10.1371/journal.pmed.1004677)
Supplement: S11 Table — (DOCX) [file pmed.1004677.s011.docx]

**S11 Table.** Subgroup analyses of risk of autoimmune disease associated with antibiotic exposure during early infancy according to **sex**

| **Sex** | **Outcome** | **exposure** | **No_Patients** | **No_Events** | **IRper100000PY** | **aHR** | **95% CI** |
| --- | --- | --- | --- | --- | --- | --- | --- |
| Male | T1D | Exposed | 761858 | 191 | 2.99 | 0.97 | 0.79 to 1.18 |
|  |  | Unexposed | 627525 | 139 | 2.80 |  |  |
|  | JIA | Exposed | 761858 | 177 | 2.77 | 1.18 | 0.96 to1.45 |
|  |  | Unexposed | 627525 | 124 | 2.50 |  |  |
|  | UC | Exposed | 761858 | 52 | 0.81 | 1.01 | 0.68 to 1.51 |
|  |  | Unexposed | 627525 | 38 | 0.76 |  |  |
|  | CD | Exposed | 761858 | 322 | 5.04 | 1.11 | 0.94 to 1.30 |
|  |  | Unexposed | 627525 | 208 | 4.19 |  |  |
|  | SLE | Exposed | 761858 | 31 | 0.48 | 1.41 | 0.79 to 2.50 |
|  |  | Unexposed | 627525 | 14 | 0.28 |  |  |
|  | HT | Exposed | 761858 | 99 | 1.55 | 1.40 | 1.02 to 1.90 |
|  |  | Unexposed | 627525 | 53 | 1.07 |  |  |
| Female | T1D | Exposed | 645691 | 221 | 4.05 | 1.10 | 0.92 to 1.31 |
|  |  | Unexposed | 655972 | 194 | 3.71 |  |  |
|  | JIA | Exposed | 645691 | 199 | 3.64 | 1.13 | 0.94 to 1.37 |
|  |  | Unexposed | 655972 | 168 | 3.21 |  |  |
|  | UC | Exposed | 645691 | 45 | 0.82 | 0.88 | 0.60 to 1.27 |
|  |  | Unexposed | 655972 | 45 | 0.86 |  |  |
|  | CD | Exposed | 645691 | 184 | 3.37 | 0.97 | 0.81 to 1.18 |
|  |  | Unexposed | 655972 | 177 | 3.38 |  |  |
|  | SLE | Exposed | 645691 | 47 | 0.86 | 1.16 | 0.77 to 1.74 |
|  |  | Unexposed | 655972 | 34 | 0.65 |  |  |
|  | HT | Exposed | 645691 | 400 | 7.32 | 0.98 | 0.86 to 1.12 |
|  |  | Unexposed | 655972 | 352 | 6.73 |  |  |

**Abbreviation:** aHR, adjusted hazard ratio; CD, Crohn's disease; CI, confidence interval; IR, incidence rate; HT, Hashimoto’s thyroiditis; JIA, juvenile idiopathic arthritis; T1D, type 1 diabetes; PY, person-year; UC, ulcerative colitis; SLE, systemic lupus erythematosus.
