# Supplementary material for: Exposure to antibiotics during pregnancy or early infancy and the risk of autoimmune disease in children: A nationwide cohort study in Korea
Source: PLoS Med. 2025 Aug 21;22(8):e1004677. doi: 10.1371/journal.pmed.1004677 (PMC12370083; doi:10.1371/journal.pmed.1004677)
Supplement: S12 Table — (DOCX) [file pmed.1004677.s012.docx]

**S12 Table.** Subgroup analyses of risk of autoimmune disease associated with antibiotic exposure during early infancy according to antibiotic **exposure timing** during early infancy

| **Timing** | **Outcome** | **exposure** | **No_Patients** | **No_Events** | **IRper100000PY** | **aHR** | **95% CI** |
| --- | --- | --- | --- | --- | --- | --- | --- |
| <2 months | T1D | Exposed | 599360 | 187 | 3.77 | 1.16 | 0.93 to 1.44 |
|  |  | Unexposed | 1308040 | 335 | 3.24 |  |  |
|  | JIA | Exposed | 599360 | 153 | 3.09 | 1.09 | 0.86 to 1.39 |
|  |  | Unexposed | 1308040 | 297 | 2.87 |  |  |
|  | UC | Exposed | 599360 | 48 | 0.97 | 1.14 | 0.74 to 1.75 |
|  |  | Unexposed | 1308040 | 83 | 0.80 |  |  |
|  | CD | Exposed | 599360 | 240 | 4.84 | 1.22 | 0.99 to 1.48 |
|  |  | Unexposed | 1308040 | 397 | 3.83 |  |  |
|  | SLE | Exposed | 599360 | 29 | 0.59 | 1.05 | 0.56 to 1.96 |
|  |  | Unexposed | 1308040 | 49 | 0.47 |  |  |
|  | HT | Exposed | 599360 | 236 | 4.76 | 1.30 | 1.07 to 1.58 |
|  |  | Unexposed | 1308040 | 405 | 3.91 |  |  |
| 3-4 months | T1D | Exposed | 628456 | 175 | 3.29 | 1.00 | 0.79 to 1.28 |
|  |  | Unexposed | 1225373 | 324 | 3.29 |  |  |
|  | JIA | Exposed | 628456 | 167 | 3.14 | 1.09 | 0.85 to 1.41 |
|  |  | Unexposed | 1225373 | 284 | 2.88 |  |  |
|  | UC | Exposed | 628456 | 42 | 0.79 | 1.15 | 0.70 to 1.89 |
|  |  | Unexposed | 1225373 | 78 | 0.79 |  |  |
|  | CD | Exposed | 628456 | 228 | 4.28 | 1.06 | 0.85 to 1.33 |
|  |  | Unexposed | 1225373 | 378 | 3.84 |  |  |
|  | SLE | Exposed | 628456 | 37 | 0.69 | 1.26 | 0.67 to 2.37 |
|  |  | Unexposed | 1225373 | 37 | 0.48 |  |  |
|  | HT | Exposed | 628456 | 243 | 4.56 | 1.16 | 0.94 to 1.44 |
|  |  | Unexposed | 1225373 | 385 | 3.91 |  |  |
| 5-6 months | T1D | Exposed | 987878 | 292 | 3.49 | 1.12 | 0.92 to 1.36 |
|  |  | Unexposed | 1226215 | 326 | 3.31 |  |  |
|  | JIA | Exposed | 987878 | 267 | 3.19 | 1.03 | 0.85 to 1.26 |
|  |  | Unexposed | 1226215 | 280 | 2.84 |  |  |
|  | UC | Exposed | 987878 | 65 | 0.78 | 0.79 | 0.52 to 1.20 |
|  |  | Unexposed | 1226215 | 80 | 0.81 |  |  |
|  | CD | Exposed | 987878 | 359 | 4.29 | 1.05 | 0.88 to 1.26 |
|  |  | Unexposed | 1226215 | 372 | 3.78 |  |  |
|  | SLE | Exposed | 987878 | 52 | 0.62 | 1.17 | 0.73 to 1.89 |
|  |  | Unexposed | 1226215 | 48 | 0.49 |  |  |
|  | HT | Exposed | 987878 | 352 | 4.21 | 1.04 | 0.87 to 1.25 |
|  |  | Unexposed | 1226215 | 393 | 3.99 |  |  |

**Abbreviation:** aHR, adjusted hazard ratio; CD, Crohn's disease; CI, confidence interval; IR, incidence rate; HT, Hashimoto’s thyroiditis; JIA, juvenile idiopathic arthritis; T1D, type 1 diabetes; PY, person-year; UC, ulcerative colitis; SLE, systemic lupus erythematosus.
