# Supplementary material for: Exposure to antibiotics during pregnancy or early infancy and the risk of autoimmune disease in children: A nationwide cohort study in Korea
Source: PLoS Med. 2025 Aug 21;22(8):e1004677. doi: 10.1371/journal.pmed.1004677 (PMC12370083; doi:10.1371/journal.pmed.1004677)
Supplement: S13 Table — (DOCX) [file pmed.1004677.s013.docx]

**S13 Table.** Sensitivity analyses of risk of autoimmune disease associated with antibiotic exposure during **pregnancy**

| **Sensitivity**  **analysis^*^** | **Outcome** | **exposure** | **No_Patients** | **No_Events** | **IR/100000PY** | **aHR** | **95% CI** |
| --- | --- | --- | --- | --- | --- | --- | --- |
| **Sensitivity**  **analysis 1** | T1D | Exposed | 749957 | 208 | 3.83 | 1.10 | 0.88 to 1.37 |
|  |  | Unexposed | 917870 | 241 | 3.53 |  |  |
|  | JIA | Exposed | 749957 | 181 | 3.33 | 1.04 | 0.82 to 1.32 |
|  |  | Unexposed | 917870 | 227 | 3.32 |  |  |
|  | UC | Exposed | 749957 | 82 | 1.51 | 1.10 | 0.77 to 1.59 |
|  |  | Unexposed | 917870 | 91 | 1.33 |  |  |
|  | CD | Exposed | 749957 | 266 | 4.89 | 1.29 | 1.04 to 1.58 |
|  |  | Unexposed | 917870 | 275 | 4.02 |  |  |
|  | SLE | Exposed | 749957 | 41 | 0.75 | 0.81 | 0.60 to 1.09 |
|  |  | Unexposed | 917870 | 67 | 0.98 |  |  |
|  | HT | Exposed | 749957 | 265 | 4.87 | 1.13 | 0.93 to 1.38 |
|  |  | Unexposed | 917870 | 304 | 4.45 |  |  |
| **Sensitivity**  **analysis 2** | T1D | Exposed | 1285075 | 375 | 3.98 | 1.12 | 0.94 to 1.33 |
|  |  | Unexposed | 1018952 | 279 | 3.66 |  |  |
|  | JIA | Exposed | 1285075 | 322 | 3.42 | 1.03 | 0.86 to 1.24 |
|  |  | Unexposed | 1018952 | 260 | 3.41 |  |  |
|  | UC | Exposed | 1285075 | 132 | 1.40 | 1.03 | 0.76 to 1.40 |
|  |  | Unexposed | 1018952 | 97 | 1.27 |  |  |
|  | CD | Exposed | 1285075 | 446 | 4.74 | 1.19 | 1.01 to 1.41 |
|  |  | Unexposed | 1018952 | 301 | 3.95 |  |  |
|  | SLE | Exposed | 1285075 | 74 | 0.79 | 0.70 | 0.49 to 1.01 |
|  |  | Unexposed | 1018952 | 69 | 0.91 |  |  |
|  | HT | Exposed | 1285075 | 455 | 4.83 | 1.05 | 0.90 to 1.23 |
|  |  | Unexposed | 1018952 | 341 | 4.48 |  |  |
| **Sensitivity**  **analysis 3** | T1D | Exposed | 432352 | 134 | 3.96 | 1.25 | 0.94 to 1.67 |
|  |  | Unexposed | 369147 | 100 | 3.41 |  |  |
|  | JIA | Exposed | 432352 | 128 | 3.78 | 1.01 | 0.87 to 1.17 |
|  |  | Unexposed | 369147 | 93 | 3.17 |  |  |
|  | UC | Exposed | 432352 | 38 | 1.12 | 0.73 | 0.42 to 1.26 |
|  |  | Unexposed | 369147 | 33 | 1.13 |  |  |
|  | CD | Exposed | 432352 | 138 | 4.08 | 1.00 | 0.76 to 1.32 |
|  |  | Unexposed | 369147 | 123 | 4.20 |  |  |
|  | SLE | Exposed | 432352 | 21 | 0.62 | 0.56 | 0.31 to 1.02 |
|  |  | Unexposed | 369147 | 26 | 0.89 |  |  |
|  | HT | Exposed | 432352 | 182 | 5.38 | 1.08 | 0.85 to 1.38 |
|  |  | Unexposed | 369147 | 141 | 4.81 |  |  |
| **Sensitivity**  **analysis 4** | T1D | Exposed | 1259596 | 360 | 3.88 | 1.15 | 0.96 to 1.38 |
|  |  | Unexposed | 988232 | 257 | 3.46 |  |  |
|  | JIA | Exposed | 1259596 | 309 | 3.33 | 1.01 | 0.84 to 1.21 |
|  |  | Unexposed | 988232 | 252 | 3.39 |  |  |
|  | UC | Exposed | 1259596 | 127 | 1.37 | 0.98 | 0.72 to 1.32 |
|  |  | Unexposed | 988232 | 96 | 1.29 |  |  |
|  | CD | Exposed | 1259596 | 439 | 4.73 | 1.15 | 0.98 to 1.36 |
|  |  | Unexposed | 988232 | 300 | 4.03 |  |  |
|  | SLE | Exposed | 1259596 | 61 | 0.66 | 0.72 | 0.48 to 1.06 |
|  |  | Unexposed | 988232 | 60 | 0.81 |  |  |
|  | HT | Exposed | 1259596 | 419 | 4.51 | 1.12 | 0.95 to 1.32 |
|  |  | Unexposed | 988232 | 298 | 4.01 |  |  |

**Abbreviation:** aHR, adjusted hazard ratio; CD, Crohn's disease; CI, confidence interval; IR, incidence rate; HT, Hashimoto’s thyroiditis; JIA, juvenile idiopathic arthritis; T1D, type 1 diabetes; PY, person-year; UC, ulcerative colitis; SLE, systemic lupus erythematosus.

**^*^Explanation of the 4 respective sensitivity analyses;**

Sensitivity 1: We modified the definition of exposure to two or more prescriptions of systemic antibiotics within the same assessment window.

Sensitivity 2: We conducted an analysis excluding multifetal gestations, restricting to singleton birth.

Sensitivity 3: We limited the study population to children who were breastfed (both fully and partially).

Sensitivity 4: We narrowed the study population to children whose mothers had not been diagnosed with autoimmune diseases.
