# Supplementary material for: Exposure to antibiotics during pregnancy or early infancy and the risk of autoimmune disease in children: A nationwide cohort study in Korea
Source: PLoS Med. 2025 Aug 21;22(8):e1004677. doi: 10.1371/journal.pmed.1004677 (PMC12370083; doi:10.1371/journal.pmed.1004677)
Supplement: S14 Table — (DOCX) [file pmed.1004677.s014.docx]

**S14 Table.** Sensitivity analyses of risk of autoimmune disease associated with antibiotic exposure during **early infancy**

| **Sensitivity**  **analysis^*^** | **Outcome** | **exposure** | **No_Patients** | **No_Events** | **IRper100000PY** | **aHR** | **95% CI** |
| --- | --- | --- | --- | --- | --- | --- | --- |
| **Sensitivity**  **analysis 1** | T1D | Exposed | 971270 | 283 | 3.46 | 1.02 | 0.89 to 1.16 |
|  |  | Unexposed | 1188577 | 311 | 3.29 |  |  |
|  | JIA | Exposed | 971270 | 260 | 3.18 | 1.14 | 0.99 to 1.31 |
|  |  | Unexposed | 1188577 | 268 | 2.84 |  |  |
|  | UC | Exposed | 971270 | 67 | 0.82 | 0.94 | 0.72 to 1.23 |
|  |  | Unexposed | 1188577 | 75 | 0.80 |  |  |
|  | CD | Exposed | 971270 | 348 | 4.26 | 1.07 | 0.95 to 1.21 |
|  |  | Unexposed | 1188577 | 358 | 3.79 |  |  |
|  | SLE | Exposed | 971270 | 55 | 0.68 | 1.19 | 0.86 to 1.66 |
|  |  | Unexposed | 1188577 | 45 | 0.47 |  |  |
|  | HT | Exposed | 971270 | 354 | 4.33 | 0.97 | 0.86 to 1.10 |
|  |  | Unexposed | 1188577 | 373 | 3.95 |  |  |
| **Sensitivity**  **analysis 2** | T1D | Exposed | 1357617 | 402 | 3.51 | 1.04 | 0.87 to 1.25 |
|  |  | Unexposed | 1241393 | 327 | 3.31 |  |  |
|  | JIA | Exposed | 1357617 | 369 | 3.22 | 1.13 | 0.94 to 1.36 |
|  |  | Unexposed | 1241393 | 282 | 2.85 |  |  |
|  | UC | Exposed | 1357617 | 93 | 0.81 | 0.95 | 0.66 to 1.36 |
|  |  | Unexposed | 1241393 | 79 | 0.80 |  |  |
|  | CD | Exposed | 1357617 | 483 | 4.22 | 1.05 | 0.89 to 1.23 |
|  |  | Unexposed | 1241393 | 377 | 3.81 |  |  |
|  | SLE | Exposed | 1357617 | 72 | 0.63 | 1.34 | 0.86 to 2.09 |
|  |  | Unexposed | 1241393 | 48 | 0.49 |  |  |
|  | HT | Exposed | 1357617 | 496 | 4.33 | 1.12 | 0.95 to 1.31 |
|  |  | Unexposed | 1241393 | 391 | 3.95 |  |  |
| **Sensitivity**  **analysis 3** | T1D | Exposed | 467379 | 135 | 3.27 | 1.12 | 0.83 to 1.50 |
|  |  | Unexposed | 475289 | 123 | 3.06 |  |  |
|  | JIA | Exposed | 467379 | 140 | 3.39 | 1.27 | 0.95 to 1.71 |
|  |  | Unexposed | 475289 | 104 | 2.59 |  |  |
|  | UC | Exposed | 467379 | 33 | 0.80 | 1.15 | 0.63 to 2.11 |
|  |  | Unexposed | 475289 | 31 | 0.77 |  |  |
|  | CD | Exposed | 467379 | 177 | 4.29 | 1.05 | 0.82 to 1.35 |
|  |  | Unexposed | 475289 | 167 | 4.15 |  |  |
|  | SLE | Exposed | 467379 | 29 | 0.70 | 1.19 | 0.87 to 1.68 |
|  |  | Unexposed | 475289 | 14 | 0.35 |  |  |
|  | HT | Exposed | 467379 | 196 | 4.75 | 1.24 | 0.97 to 1.58 |
|  |  | Unexposed | 475289 | 172 | 4.28 |  |  |
| **Sensitivity**  **analysis 4** | T1D | Exposed | 1343282 | 381 | 3.35 | 1.06 | 0.88 to 1.27 |
|  |  | Unexposed | 1224205 | 316 | 3.23 |  |  |
|  | JIA | Exposed | 1343282 | 362 | 3.18 | 1.13 | 0.94 to 1.36 |
|  |  | Unexposed | 1224205 | 273 | 2.79 |  |  |
|  | UC | Exposed | 1343282 | 93 | 0.82 | 0.93 | 0.65 to 1.34 |
|  |  | Unexposed | 1224205 | 78 | 0.80 |  |  |
|  | CD | Exposed | 1343282 | 477 | 4.19 | 1.05 | 0.89 to 1.24 |
|  |  | Unexposed | 1224205 | 373 | 3.81 |  |  |
|  | SLE | Exposed | 1343282 | 72 | 0.63 | 1.43 | 0.91 to 2.26 |
|  |  | Unexposed | 1224205 | 45 | 0.46 |  |  |
|  | HT | Exposed | 1343282 | 470 | 4.13 | 1.15 | 0.97 to 1.35 |
|  |  | Unexposed | 1224205 | 362 | 3.70 |  |  |

**Abbreviation:** aHR, adjusted hazard ratio; CD, Crohn's disease; CI, confidence interval; IR, incidence rate; HT, Hashimoto’s thyroiditis; JIA, juvenile idiopathic arthritis; T1D, type 1 diabetes; PY, person-year; UC, ulcerative colitis; SLE, systemic lupus erythematosus.

**^*^Explanation of the 4 respective sensitivity analyses;**

Sensitivity 1: We modified the definition of exposure to two or more prescriptions of systemic antibiotics within the same assessment window.

Sensitivity 2: We conducted an analysis excluding multifetal gestations, restricting to singleton birth.

Sensitivity 3: We limited the study population to children who were breastfed (both fully and partially).

Sensitivity 4: We narrowed the study population to children whose mothers had not been diagnosed with autoimmune diseases.
