# Supplementary material for: Exposure to antibiotics during pregnancy or early infancy and the risk of autoimmune disease in children: A nationwide cohort study in Korea
Source: PLoS Med. 2025 Aug 21;22(8):e1004677. doi: 10.1371/journal.pmed.1004677 (PMC12370083; doi:10.1371/journal.pmed.1004677)
Supplement: S1 Protocol — (DOCX) [file pmed.1004677.s015.docx]

**S1 Protocol.** Summary Protocol

| **Protocol Title:** | Exposure to antibiotics during pregnancy or early infancy and the risk of autoimmune disease in children: A nationwide cohort study in Korea |
| --- | --- |
| **Study Objective:** | To investigate the association between antibiotic exposure during pregnancy and early infancy and the risk of autoimmune diseases. |
| **Study type:** | Observational retrospective cohort study |
| **Study Design:** | A retrospective nationwide cohort study using the National Health Insurance Service (NHIS) database of South Korea, which covers the entire South Korean population. |
| **Study Population:** | Mother-child pairs identified from the National Health Insurance Service database of South Korea between 2008 and 2021. All live births will be included as eligible population for both pregnancy and infancy analyses. |
| **Intervention:** | Exposure to systemic antibiotics [ATC code: J01] during pregnancy (from the last menstrual period to the end of pregnancy) or during early infancy (first 6 months of life). |
| **Sample Size:** | A total of approximately 2.4 million and 2.7 million children will be included for the pregnancy and infancy exposure analyses, respectively, among nearly 4 million children identified in the nationwide mother-child linked database. |
| **Study Endpoints:** | The outcomes of interest will include six autoimmune diseases: 1) type 1 diabetes, 2) juvenile idiopathic arthritis, 3) ulcerative colitis, 4) Crohn’s disease, 5) systemic lupus erythematosus, and 6) Hashimoto’s thyroiditis. All autoimmune diseases will require at least one inpatient or at least two outpatient diagnoses within one year to improve the specificity of the outcome. For the pregnancy analysis, children will be followed from the day they are born (delivery date) until the occurrence of the outcome, death, or December 31, 2021, ensuring at least a one-year follow-up period for all children. In the infancy analysis, children will be followed from 180 days after birth until the occurrence of the outcome, death, or December 31, 2021. |
| **Statistical Methods:** | Propensity score matching will be applied to enhance comparability and adjust for baseline differences between the exposed and unexposed groups.  Additionally, sibling-matched analyses were performed to control shared familial and genetic factors.  Cox proportional hazards models will be used to estimate hazard ratios (HRs) and 95% confidence intervals (CIs) for the risk of autoimmune diseases associated with antibiotic exposure. |
| **Contacts and Locations:** | **Principal Investigator:** Ju-Young Shin, PhD, Sungkyunkwan University  **Locations:** Sungkyunkwan University, Suwon, Gyeonggi-do 16419 Republic of Korea  **Sponsors and Collaborators:** National Research Foundation of Korea and Health Fellowship Foundation (2023) |

***Tabular Overview of Analytical Changes**

|  | Original Protocol  (IRB-Approved) | Analysis performed  (Revised Manuscript) | Reason for Change |
| --- | --- | --- | --- |
| **Analytic Method** | 1:1 Propensity Score Matching (greedy nearest neighbor, without replacement) | Stabilized Inverse Probability of Treatment Weighting (IPTW) | Reviewer suggestion; to include a larger proportion of eligible subjects, particularly given the comparable sizes of the antibiotics-exposed and unexposed groups. |
